# An AI-assisted designed supramolecularly engineered nanoplatform reverses pigmentation by triggering an ineffective compensatory melanin production program

**DOI:** 10.1016/j.bioactmat.2026.01.027

**Published:** 2026-01-24

**Authors:** Tianqi Liu, Liang Chen, Xiaoyu Zhao, Min Xie, Ling Xie, Mi Wang, Zhenyuan Wang, Jiaheng Zhang

**Affiliations:** a School of Biomedical Engineering, Harbin Institute of Technology, Shenzhen, 518055, China; b Sauvage Laboratory for Smart Materials, School of Materials Science and Engineering, Harbin Institute of Technology, Shenzhen, 518055, China; c Scientific Research Laboratory, Shanghai Le-Surely Biotechnology Co., Ltd, Shanghai, China; d SASELOMO Research Institute and Biological Laboratory, Shanghai Chuanmei Industrial Co., Ltd, Shanghai, China; e School of Biomedical Sciences, LKS Faculty of Medicine, The University of Hong Kong, Hong Kong, Hong Kong SAR, China; f Shenzhen Shinehigh Innovation Technology Co., Ltd., Shenzhen 518055, China; g Gd Shinesky Medical Supplies Health Technology Co., Ltd., Shaoguan 512029, China

**Keywords:** AI-Assisted design, Supramolecular assembly, Hyperpigmentation, Functional inhibition, Transdermal delivery

## Abstract

The clinical applications of natural compounds are limited by their inherent physicochemical properties. This study reports a hierarchical supramolecular engineering strategy for constructing a dual-assembly nanosystem for the treatment of skin hyperpigmentation. Using an AI-assisted computational screening model, tranexamic acid was identified as a suitable molecular partner of the hydrophobic and active, baicalin. Subsequent dual assembly processes yielded a stable hybrid nanoplatform (DHBTC) that enhanced the solubility and delivery efficiency of baicalin. Single-cell transcriptomics revealed an unexpected mechanism of "functional inhibition"; despite the depigmenting efficacy of DHBTC, the melanogenesis-related gene network in melanocytes was upregulated. This was identified as compensatory transcriptional feedback triggered by drug-induced autophagy. DHBTC functionally inhibits pigment accumulation by accelerating melanosome degradation, which elicits ineffective transcriptional activation as the cell attempts to restore homeostasis. Furthermore, the platform remodeled the cutaneous immune microenvironment toward an anti-inflammatory state. This study presents a strategy for designing drug delivery systems, from computational prediction to supramolecular assembly, and describes a therapeutic mechanism based on the modulation of post-translational and organellar homeostasis.

## Highlights

• An AI-driven reverse design strategy guided the hierarchical supramolecular assembly, yielding the DHBTC nanoplatform that addresses key delivery challenges of baicalin, including its stability, solubility, and transdermal permeability.
• Single-cell transcriptomics revealed a novel functional inhibitory mechanism, wherein DHBTC accelerates autophagy-mediated melanosome clearance, triggering a compensatory yet ineffectual transcriptional upregulation of the melanogenesis program.
• DHBTC reverses hyperpigmentation via a dual therapeutic strategy: blocking pigment accumulation at the post-translational level while simultaneously remodeling the skin immune microenvironment toward a homeostatic anti-inflammatory state.

## Graphical abstract

This study presents a platform strategy for drug delivery system design, spanning from computational prediction to supramolecular construction. The strategy first employed an AI-driven reverse design to screen and predict tranexamic acid as the ideal molecular partner for the hydrophobic baicalin. Subsequently, through hierarchical supramolecular engineering, the components were self-assembled and encapsulated with cyclodextrin to build a novel nanoplatform (DHBTC). This platform overcame key biopharmaceutical barriers, increasing baicalin's solubility 608-fold and enhancing penetration. Mechanistically, it effectively reversed hyperpigmentation by accelerating autophagy-mediated melanosome clearance and remodeling the skin's immune microenvironment toward an anti-inflammatory state.

**Figure:**
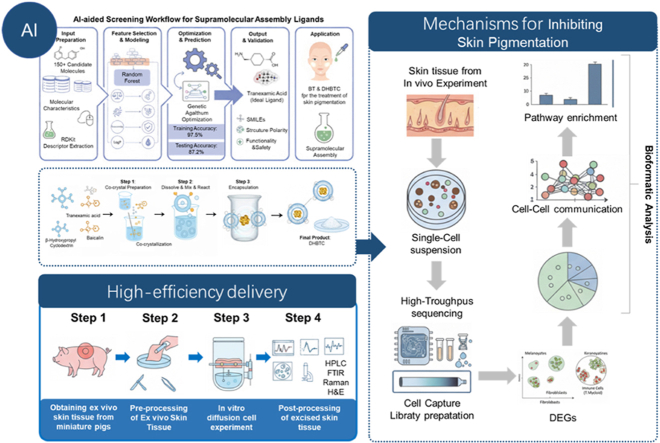

## 1 Introduction

Modern drug discovery faces a pervasive pharmaceutical challenge; a vast number of highly active lead compounds fail to become viable drugs because of their poor aqueous solubility and low biological membrane permeability [1,2]. This biopharmaceutical bottleneck is particularly pronounced in transdermal drug delivery. As the primary defense system of the body, the skin possesses an outermost stratum corneum (SC) composed of tightly packed corneocytes and lipid bilayers, forming a formidable barrier that substantially restricts the penetration of most exogenous molecules [[3], [4], [5], [6], [7]]. Drug delivery challenges are further exacerbated when a therapeutic strategy requires the co-delivery of two or more active molecules with disparate physicochemical properties, such as one being hydrophobic and the other hydrophilic [[8], [9], [10]]. Conventional single-formulation platforms are often incapable of simultaneously satisfying these contradictory delivery requirements, which is a long-standing technical hurdle in dermatological drug delivery [2,[11], [12], [13]].

Baicalin (BA), a flavonoid derived from traditional medicinal herbs, is a clinically valuable example of this challenge [[14], [15], [16], [17], [18]]. BA has potent antioxidant, anti-inflammatory, and anti-photoaging activities, demonstrating its immense therapeutic potential for treating skin hyperpigmentation, inflammatory dermatoses, and other skin disorders [[19], [20], [21], [22], [23], [24]]. However, the clinical translation of BA has long been impeded by its intrinsic physicochemical drawbacks [25]. As a typical Biopharmaceutics Classification System Class IV drug, it's extremely low water solubility and poor permeability result in negligible transdermal penetration, making it difficult to achieve therapeutic concentrations in the target tissue [[26], [27], [28]]. Therefore, the development of an innovative strategy to overcome this delivery barrier and fully elucidate the biological potential of BA is of substantial scientific and clinical importance [29,30].

To address the delivery challenges of BA, various formulations, such as microemulsions, solid lipid nanoparticles, and nanostructured lipid carriers, have been developed [[31], [32], [33], [34]]. While these studies have achieved a degree of success in enhancing BA delivery, they share a fundamental limitation; the strategies rely on "passive encapsulation," which fails to alter the inherent unfavorable properties of the molecule at a fundamental level [[35], [36], [37], [38]]. Moreover, when co-delivery is involved, the choice of a partner molecule is typically based on its established clinical application rather than its intrinsic physicochemical synergy with BA to form a stable and efficient delivery entity [25,[39], [40], [41]]. This reliance on empirical, trial-and-error screening, restricts the discovery of unclear, superior molecular combinations, representing a methodological bottleneck in the field [42,43].

Supramolecular self-assembly strategies offer a powerful solution to these challenges. By leveraging non-covalent interactions-such as hydrogen bonding, π-π stacking, and host-guest interactions-supramolecular engineering enables the precise organization of drug molecules with disparate properties into thermodynamically stable nanostructures [44,45]. This approach not only effectively resolves technical bottlenecks related to solubility enhancement and co-delivery but also imparts unique advantages to the delivery system, including dynamic reversibility and intelligent responsive release [46,47]. Consequently, constructing functional supramolecular assemblies represents a promising avenue for revitalizing insoluble natural active ingredients.

In this study, we propose a paradigm shift from "forward screening" to "AI-guided inverse design," aiming to answer a core scientific question: How can we utilize AI-guided supramolecular engineering to bridge the "high bioactivity–low bioavailability" gap of natural products like Baicalin, and thereby reveal their unique intracellular homeostatic regulation mechanisms? Moving beyond the traditional inert carrier concept, we employed artificial intelligence (AI) as a rational discovery tool to identify, from a vast chemical space, a hydrophilic molecule (Tranexamic Acid, TA) that acts not merely as a solubilizer but as a synergistic "active excipient." Guided by this prediction, we constructed a hierarchical supramolecular engineering platform (DHBTC). This system resolves the delivery paradox at the molecular level through hydrogen-bond-mediated co-assembly and host-guest encapsulation, achieving a 608-fold increase in solubility. Furthermore, utilizing single-cell transcriptomics and functional validation, we elucidate a novel therapeutic mechanism: the nanoplatform functionally inhibits pigmentation by inducing autophagy-mediated melanosome clearance-triggering an ineffective transcriptional compensation-while simultaneously remodeling the cutaneous immune microenvironment toward a homeostatic state. This work presents a holistic strategy for designing functional drug delivery systems, integrating computational prediction, supramolecular assembly, and mechanistic modulation of cellular homeostasis.

## 2 Results

### 2.1 AI-driven supramolecular structure design and reliability verification

An inverse design strategy driven by AI was used to identify a molecular partner of BA that possesses both complementary biological activity and ideal physicochemical properties. We constructed and trained a back-propagation neural network model based on a dataset of 159 experimental entries. Following feature selection and importance ranking using a Random Forest algorithm (Supplementary Fig. 1a–c), the molecular SMILES string, number of hydrogen bond donors, molecular weight, tyrosinase inhibitory potential, and topological polar surface area were selected as the core predictive variables.

**Fig. 1:**
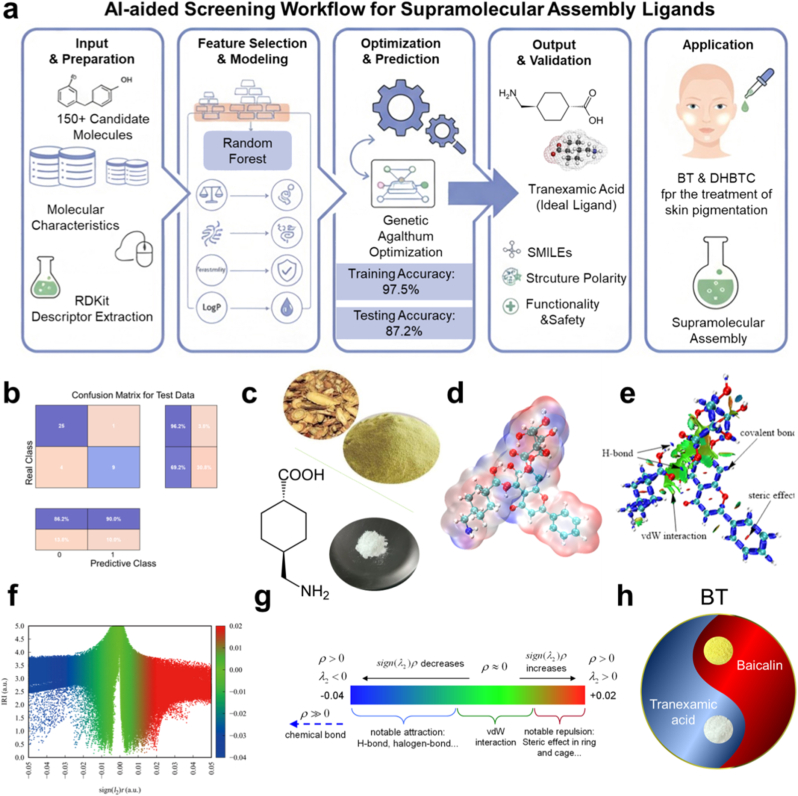
**BT's Artificial Intelligence (AI)-driven Design, Theoretical Verification, and Self-assembly Strategy. a**, Flowchart of the AI-assisted screening process for identifying the optimal molecular partner for baicalin (BA). **b**, Confusion matrix of the AI model on the validation set. **c**, Physical image of baicalin (top). Chemical structure and physical image of tranexamic acid (TA) (bottom). **d**, Electrostatic potential (ESP) surface plot of the BA-TA (BT) assembly. Red and blue areas represent positive and negative electrostatic potential, respectively. **e**, Visualization of the interaction region indicator (IRI) analysis between BT. **f, g**, Scatter plots of the IRI interaction analysis and corresponding color bars, where sign(λ^2^)ρ is the product of the electron density and the second Hessian eigenvalue. **h**, Conceptual schematic of the BT supramolecular assembly formed by multipoint hydrogen bonding between BA and TA.

The overall workflow for the AI-assisted screening is illustrated in Fig. 1a. The model performance evaluation demonstrated strong classification performance for the training set of 120 entries (Supplementary Fig. 1b) and achieved a prediction accuracy of 87.2 % for a test set of 39 entries (Supplementary Fig. 1c), confirming its robust generalization ability and predictive reliability. The confusion matrices (Fig. 1b, Supplementary Fig. 1d) further validated the classification performance of the model, indicating that the AI model could accurately identify the characteristics of ideal ligands for forming efficient and stable supramolecular systems with BA.

Subsequently, we integrated this AI model with a genetic algorithm to perform a global optimization search under predefined physicochemical and biological constraints. After approximately 60 iterations, the performance curve of the algorithm converges (Supplementary Fig. 1e), ultimately identifying tranexamic acid (TA) as the optimal bioactive partner of BA (Fig. 1c).

To validate the reliability of AI prediction from first principles, and elucidate the nature of the interaction between BA and TA, we performed density functional theory (DFT) calculations. Electrostatic potential (ESP) analysis (Fig. 1d) visually demonstrated the charge complementarity between the two molecules. The phenolic hydroxyl regions of BA exhibited a significant negative potential (nucleophilic sites), whereas the carboxyl and ammonium protons of TA showed a strong positive potential (electrophilic sites). This provides an electrostatic driving force for the formation of strong hydrogen bonds. The independent gradient model (IGM) and interaction region indicator (IRI) analyses further visualized the non-covalent interactions (NCI) between the molecules. Extensive green isosurfaces are clearly visible between the multiple phenolic hydroxyl groups of BA, and the carboxyl and amino groups of TA (Fig. 1e). According to the IRI theory (Fig. 1f and g; Supplementary Fig. 1f and g), these represent strong hydrogen bonding and van der Waals attractive forces. These simulation results provide robust theoretical support for AI screening and precisely depict the interaction pattern of a BATABT assembly stabilized by a multi-point, synergistic hydrogen-bonding network (Fig. 1h).

### 2.2 Preparation, characterization, and Performance Analysis of the BT assembly

**Fig. 2:**
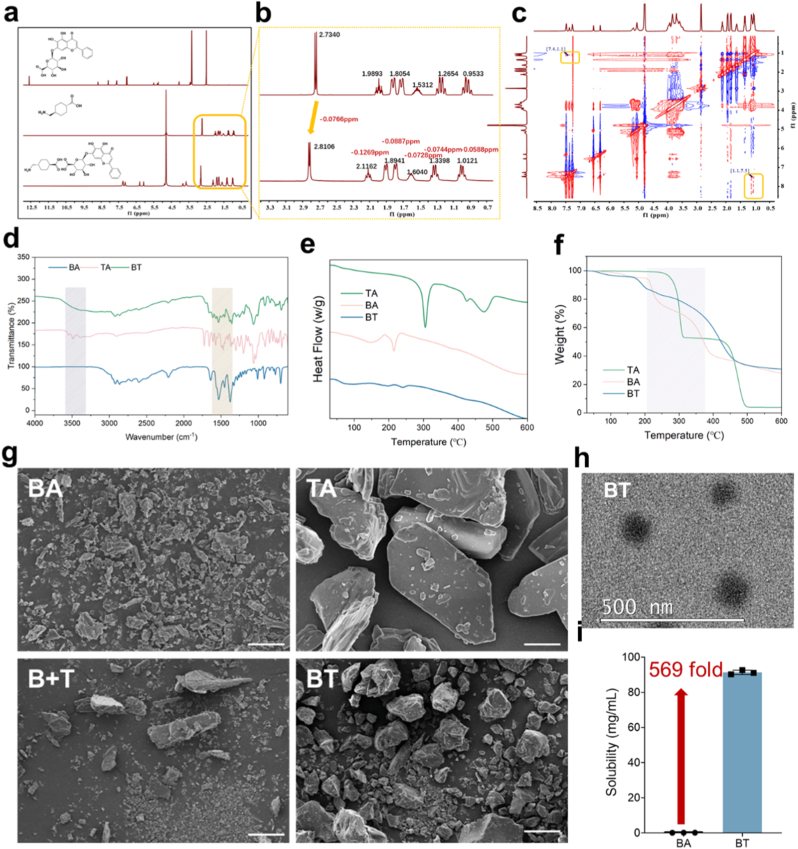
**Preparation, Characterization, and Performance Analysis of the baicalin-tranexamic acid (BT) Supramolecular Assembly**. **a**, ^1^H NMR spectra of baicalin (BA), tranexamic acid (TA), and the BT assembly. **b**, Local magnification of the spectrum in (a). **c**, The 2D NOESY spectrum of the BT assembly. **d**, Fourier-transform infrared spectroscopy (FTIR) spectra of BA, TA, and the BT assembly. **e**, Differential scanning calorimetry (DSC) curves of BA, TA, and the BT assembly. **f**, Thermogravimetric analysis (TGA) curves of BA, TA, and the BT assembly. **g**, Scanning electron microscopy (SEM) images of the solid-state powders of BA, TA, B + T, and the BT assembly. Scale bar = 10 μm **h**, Transmission electron microscopy (TEM) image of nanoparticles formed by the self-assembly of the BT assembly in water. Scale bar = 500 nm. **i**, Comparison of the apparent aqueous solubility of BA as a raw drug versus within the BT assembly.

Based on theoretical predictions, a BT assembly was prepared at various molar ratios via solvent evaporation, yielding a physically uniform, pale-yellow solid powder (Supplementary Fig. 2a). To confirm the successful formation of the BT assembly and elucidate the intermolecular interactions within it, a series of systematic spectroscopic characterizations were performed. As shown in Fig. 2a and b, compared with the spectrum of pure TA, the proton signals of the aliphatic skeleton within the BT assembly exhibited distinct downfield shifts. Specifically, the characteristic methylene proton peak adjacent to the amino group shifted from 2.73 ppm to 2.81 ppm, while other aliphatic protons in the high-field region (0.9–2.2 ppm) exhibited corresponding shifts ranging from 0.06 to 0.13 ppm (Fig. 2b–Supplementary Table 1). This deshielding effect indicates a decrease in electron density around these protons, providing spectroscopic evidence that the functional groups of TA are involved in the formation of an extensive intermolecular hydrogen-bonding network with BA. Furthermore, the two-dimensional Nuclear Overhauser Effect Spectroscopy (NOESY) spectrum (Fig. 2c) provides robust spatial evidence to corroborate the ^1^H NMR findings. As illustrated in Fig. 2c, distinct cross-peaks were observed at the coordinates (7.4, 1.1) ppm and (1.1, 7.5) ppm. These signals reveal a strong correlation between the aromatic protons of the BA skeleton and the aliphatic methylene protons of the TA cyclohexane ring, indicating that the two molecules are in close spatial proximity, thereby confirming the formation of the supramolecular assembly. Fourier-transform infrared spectroscopy (FTIR) provided complementary evidence (Fig. 2d). The sharp O-H and N-H stretching vibration peaks of BA and TA at ∼3400 cm^−1^ merged, broadened, and red-shifted to a single wide absorption band at ∼3250 cm^−1^ in the BT assembly, a hallmark of an extensive intermolecular hydrogen-bonding network. Differential scanning calorimetry (DSC) curves (Fig. 2e) revealed that the sharp crystalline melting endotherms of BA (∼223 °C) and TA (>300 °C) completely disappeared in the BT assembly. Instead, a single, distinct glass transition temperature (Tg) emerged at approximately 85 °C, demonstrating that BT is a uniform and thermodynamically stable system. Thermogravimetric analysis (TGA) curves (Fig. 2f, Supplementary Fig. 2b) showed that the onset decomposition temperature (T onset) of BT (∼240 °C) was intermediate between that of the two raw materials, and its thermal weight loss profile exhibited a single-stage pattern completely different from that of a physical mixture, further confirming the formation of a new solid-state phase.

This remodeling of the physicochemical properties, induced by supramolecular assembly, endows the BT system with the ability to self-assemble in the aqueous phase. Scanning electron microscopy (SEM) images (Fig. 2g) showed the morphological transition from the highly crystalline forms of the raw materials (BA and TA) and their simple physical mixture (B + T) to the irregular, more fused surface morphology of the BT assembly, which is consistent with the state revealed by DSC results. Transmission electron microscopy (TEM) observations (Fig. 2h) showed that BT spontaneously self-assembled into uniform, spherical nanoparticles when dispersed in water. Dynamic light scattering (DLS) (Supplementary Table 2) was used to systematically characterize the BT nanoparticles formed at different molar ratios. The 1:1 M ratio of BT yielded nanoparticles with the most favorable physicochemical properties: a mean hydrodynamic diameter of 156.48 nm and polydispersity index (PDI) of 0.277. Despite a near-neutral surface zeta potential (−3.0 mV), stability tests confirmed that the BT nano-suspension remained clear and uniform after storage at room temperature, 4 °C, and 45 °C, demonstrating excellent physical stability (Supplementary Fig. 2c). Notably, the formation of this assembly substantially improved the solubility of BA; its aqueous solubility was enhanced by 569-fold compared with that of the raw drug (Fig. 2i). Therefore, BT (1:1) was selected for subsequent experiments.

### 2.3 Construction and Characterization of the Supramolecular Hybrid Nanoplatform (DHBTC)

**Fig. 3:**
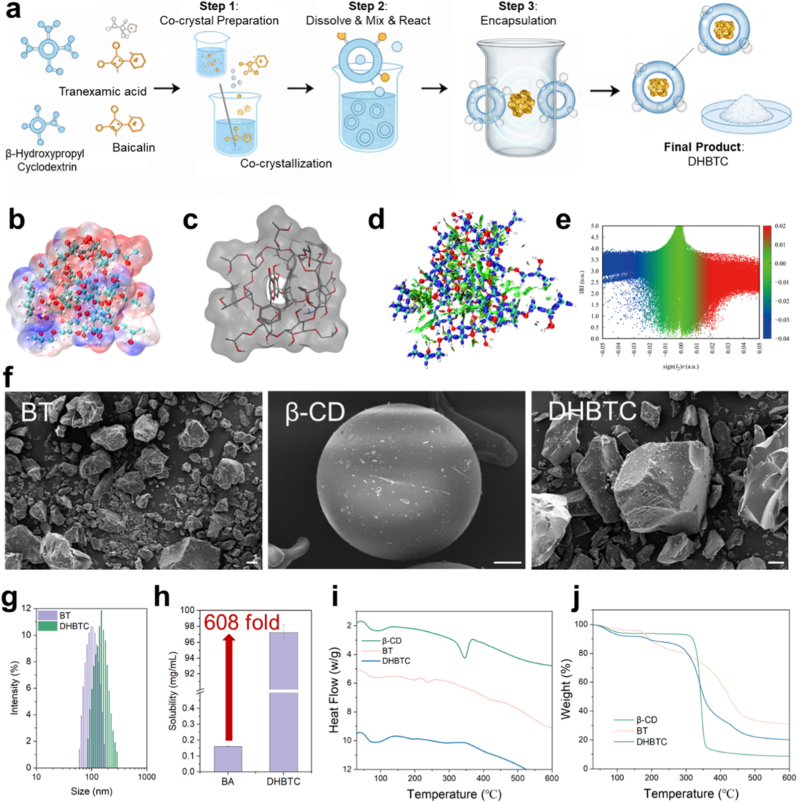
**Construction and Characterization of the Supramolecular Hybrid Nanoplatform (DHBTC). a**, Schematic illustration of the preparation of DHBTC. **b, c**, Electrostatic potential (ESP) and simulated structure diagram of DHBTC complex. **d, e**, Interaction region indicator (IRI) analysis and corresponding scatter plot for the DHBTC complex. **f**, Scanning electron microscopy (SEM) images of HP-β-CD, the baicalin-tranexamic acid (BT) assembly, and the final DHBTC product. Scale bar = 10 μm **g**, Particle size distributions of BT and DHBTC. **h**, Comparison of the apparent aqueous solubility of baicalin (BA) as a raw drug within DHBTC. **i, j**, Differential scanning calorimetry (DSC) and thermogravimetric analysis (TGA) curves of HP-β-CD, BT, and DHBTC.

To further optimize the delivery performance of the BT assembly, the biocompatible hydroxypropyl-β-cyclodextrin (HP-β-CD) was selected as a host molecule to encapsulate the BT nanocore via host-guest interactions, thereby constructing the final supramolecular assembly-cyclodextrin hybrid nanosystem (DHBTC). The preparation process is schematically illustrated in Fig. 3a. We first performed DFT calculations to theoretically predict the energy and structural bases of the secondary assembly process. The simulation results indicated that the hydrophobic portion of BA within the BT assembly preferentially inserts into the hydrophobic cavity of HP-β-CD (Fig. 3b and c), while the hydrophilic groups on the BT surface form new hydrogen bonds with the hydroxyl groups on the exterior of the HP-β-CD. IRI analysis (Fig. 3d and e) further visualized this interaction, clearly showing extensive van der Waals attractive forces (green isosurfaces) between the host and guest. The calculated negative binding energy (ΔE = −14.13 kcal/mol) indicated that this is a thermodynamically spontaneous and favorable inclusion process, predicting a stable hybrid structure formation.

The experimental characterization results were in good agreement with the theoretical predictions. SEM images (Fig. 3f) visually demonstrated the morphological transformation of the solid powder: in contrast to the irregular aggregated morphology of BT and smooth spherical shape of the raw HP-β-CD, the final product DHBTC exhibited a new, block-like structure with well-defined edges and crystal facets, indicating that the secondary assembly process led to a reconstruction of the solid-state phase. When dispersed in an aqueous phase, DLS measurements (Fig. 3g–Supplementary Table 3) provided quantitative data: the mean hydrodynamic diameter of DHBTC increased to 196.04 nm from 156.48 nm for BT, while the PDI remained low at 0.265. This increase in size is consistent with the expected encapsulation of BT by HP-β-CD. The change in zeta potential (Supplementary Table 3) further corroborated the successful coating: the surface potential of DHBTC changed from −3.0 mV for BT to −6.1 mV. This slight increase in negative charge is attributed to the exposure of the numerous hydroxyl groups on the exterior of HP-β-CD, which alters the electrochemical properties of the nanoparticle surface.

Thermal analysis provided further evidence of the formation of DHBTC as a new solid phase. In the DSC thermogram (Fig. 3i), the thermal signatures characteristic of the individual precursors (HP-β-CD and BT assembly) were absent, replaced by a unique profile. This indicated that significant intermolecular interactions led to the formation of a single thermodynamically distinct entity. This conclusion was corroborated by TGA (Fig. 3j), which revealed the thermal decomposition profile of the DHBTC intermediate into its constituent components, further supporting the successful construction of the hybrid system. The secondary assembly further enhanced BA solubility. DHBTC increased BA solubility to 97.3 mg/mL (Fig. 3h), representing an approximately 608-fold enhancement compared with that of the raw drug. Furthermore, the HP-β-CD coating significantly improved the physical stability of the nanosystem. The DHBTC nano-suspension remained clear and transparent without precipitation or aggregation after long-term storage at room temperature, 4 °C, and 45 °C (Supplementary Fig. 3), demonstrating that the HP-β-CD shell imparts excellent stability to the system.

### 2.4 DHBTC achieves efficient transdermal delivery via reversible stratum corneum fluidization

**Fig. 4:**
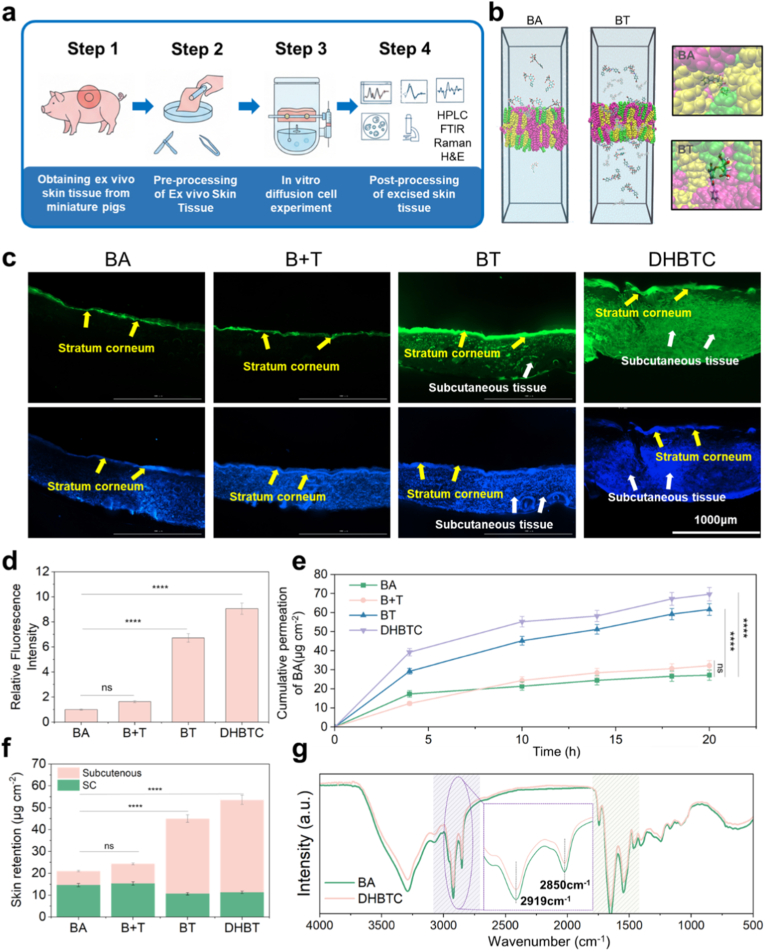
**Transdermal Delivery Performance and Mechanism of DHBTC. a**, Schematic diagram of the in vitro permeability evaluation of DHBTC. **b**, Molecular dynamics (MD) simulation showing the passage of baicalin (BA) and BA-tranexamic acid (BT) through the simulated skin lipid bilayer. **c**, Confocal laser scanning microscopy (CLSM) images of ex vivo porcine skin from different treatment groups after Franz diffusion cell experiments (BA was labeled with FITC). Scale bar = 1000 μm **d**, Semi-quantitative analysis of fluorescence intensity in (c). **e**, Cumulative permeation of BA in different treatment groups after 20 h **f**, Retention of BA in different skin layers after in vitro permeation experiments. **g**, FTIR spectra of detached skin stratum corneum before and after in vitro permeation experiment. Results are shown as mean ± SD, ns = no significant difference, ∗∗p < 0.01, ∗∗∗p < 0.001, ∗∗∗∗p < 0.0001.

To systematically evaluate the performance of DHBTC as a delivery vehicle, we conducted a multiscale investigation ranging from molecular simulations to ex vivo experiments (Fig. 4a). First, the transmembrane behavior of the BT assembly was predicted using molecular dynamics (MD) simulations within a validated SC lipid bilayer model. The simulation results revealed that compared with free BA, the BT assembly was more effectively inserted into the highly ordered lipid molecules and significantly disrupted their arrangement (Fig. 4b), thereby increasing the fluidity of the bilayer membrane.

To reveal the penetration mechanisms of BT and DHBTC at the molecular level, we performed a spectral analysis of skin tissues after different treatments. Confocal laser scanning microscopy (CLSM) was used to observe the penetration of fluorescently labeled BA into the different treatment groups (Fig. 4c). There results were significant differences in penetration between the groups. The fluorescence signals in the BA and B + T groups were weak and almost entirely confined to the skin surface layer. The fluorescence intensity and penetration depth in the BT and DHBTC groups were significantly enhanced. Semi-quantitative analysis (Fig. 4d) confirmed that the fluorescence intensities in the BT and DHBTC groups were 6.7- and 9-folds that of the BA group, respectively. The quantitative analysis provided conclusive data to support these findings. The cumulative transdermal amount of BA from the DHBTC group reached 69.7 μg/cm^2^ within 20 h (Fig. 4e), a value significantly higher than that of the BA raw drug, the physical mixture. To assess drug retention within the skin, drug extraction was performed following a permeation study. BA retention in the skin tissue was low in the BA and B + T groups, with the highest concentration in the SC. However, BA retention in the BT and DHBTC groups significantly increased, and was most concentrated in the subcutaneous tissue (Fig. 4f). This is crucial for forming a drug reservoir within the target tissue to achieve a sustained, localized therapeutic effect.

To experimentally validate the "fluidization" mechanism predicted by MD simulations at the molecular level, we performed spectroscopic analysis on the SC after treatment with the different formulations. The FTIR results (Fig. 4g) showed that compared with the BA-treated group, the absorption peaks corresponding to the asymmetric and symmetric C-H stretching vibrations of the lipid acyl chains (at ∼2919 and ∼2850 cm^−1^, respectively) in the DHBTC-treated SC shifted to higher wave numbers. This blue shift provides direct spectroscopic evidence of an increase in the conformational disorder of the lipid chains (i.e., a transition from an ordered all-trans to a disordered gauche conformation). Furthermore, no significant macroscopic changes were observed in the ex vivo skin tissue before and after the in vitro permeation experiment, or in the different treatment groups. Overall, the penetration-enhancing effect of DHBTC is an efficient and reversible physical process.

### 2.5 DHBTC exerts a powerful antioxidant function by activating the Nrf2 pathway

**Fig. 5:**
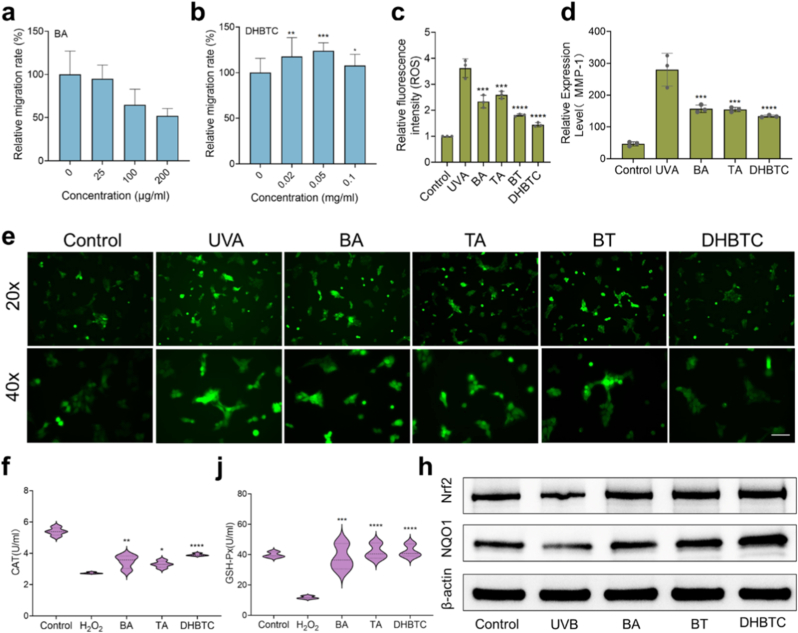
**Antioxidant, and Anti-Photoaging Functions of DHBTC. a, b**, Relative quantitative analysis of cell migration rate under the action of BA and DHBTC in HSF cell scratch wound healing assay. **c, e**, Intracellular ROS levels detected by the DCFH-DA fluorescent probe in a UVA-induced oxidative stress model. **d**, RT-qPCR analysis of MMP-1 gene expression in UVA-irradiated HSF cells. **f, j**, Activity assays for the endogenous antioxidant enzymes CAT and GSH-Px in an H_2_O_2_-induced oxidative stress model. **h**, Western blot analysis of Nrf2 and NQO1 protein expression in a UVB-irradiated cell model. Results are shown as mean ± SD, ∗p < 0.05, ∗∗p < 0.01, ∗∗∗p < 0.001, ∗∗∗∗p < 0.0001.

We comprehensively evaluated DHBTC biological effects. Before assessing the bioactivity, we first evaluated its cytotoxicity to establish a safe dosing concentration range. Based on our safety assessment results detailed below (see Section 2.9 and Fig. 9), DHBTC exhibits excellent biocompatibility at concentrations up to 0.5 mg/mL in Hacat cells. The cell scratch assay showed that BA slightly promoted cell migration at low concentrations, but exhibited a significant inhibitory effect at high concentrations (100 μg/mL) due to cytotoxicity (Fig. 5a). However, the DHBTC-treated group exhibited a significant dose-dependent pro-migratory effect and its ability to promote healing at a concentration of 0.02 mg/mL far exceeded that of BA (Fig. 5b), indicating that DHBTC has the potential to promote fibroblast migration and participate in skin tissue repair.

Given that oxidative stress is the core driver of photodamage and pigmentation, we systematically investigated the antioxidant activities of DHBTC. In a UVA-induced cellular oxidative stress model, UVA irradiation significantly increased intracellular reactive oxygen species (ROS) levels, as detected using a DCFH-DA probe. All treatment groups were able to inhibit ROS to varying degrees, among which DHBTC showed the strongest ROS scavenging ability (−61 %) (Fig. 5c–e). This suggests that the supramolecular assembly of BA and TA, and their subsequent hybridization with β-CD produced a synergistic antioxidant effect greater than the sum of the individual components. We further investigated whether this antioxidant effect stems from the modulation of the endogenous antioxidant system. In an H_2_O_2_-induced oxidative stress model, biochemical assays revealed that DHBTC was most effective at restoring the activity of stress-suppressed endogenous antioxidant enzymes, including catalase (CAT), glutathione peroxidase (GSH-Px), and superoxide dismutase (SOD) (Fig. 5f–j, Supplementary Fig. 4a). This indicates that the antioxidant mechanism of DHBTC involves direct chemical neutralization and activation of the cell defense systems for more durable and efficient protection.

To investigate the upstream regulatory mechanism underlying this biological effect, we examined the Nrf2 signaling pathway. Western blotting analysis (Fig. 5h) showed that in the UVB-irradiated cell model, DHBTC treatment significantly upregulated the expression of Nrf2 and its key downstream target protein, NAD(P)H quinone oxidoreductase 1 (NQO1). This effect was superior to that of free BA. Therefore, the potent antioxidant function of DHBTC is achieved through the activation of the Nrf2 signaling pathway.

Finally, we assessed the potential of DHBTC in combating photoaging. In a UVA-irradiated HSF cell model, DHBTC most effectively inhibited the gene expression of matrix metalloproteinase-1 (MMP-1) (−52.4 %), which is responsible for collagen degradation (Fig. 5d). Concurrently, it significantly reversed the UVA-induced downregulation of TIMP-1 (Supplementary Fig. 4b). This action synergizes with the inhibition of MMP-1 to preserve the structural integrity of the extracellular matrix. Moreover, DHBTC showed an inhibitory effect on hyaluronidase (HYAL-1), which is responsible for hyaluronan degradation, compared to that in the model group (Supplementary Fig. 4c). Collectively, these data confirmed that DHBTC is a safe and multi-functional cytoprotective agent with significant antioxidant and anti-photoaging potential.

### 2.6 Anti-inflammatory and autophagy-promoting effects of DHBTC at the cellular level

Given that inflammatory responses and disruptions in intracellular homeostasis are key drivers of pathological processes, such as skin hyperpigmentation, we further investigated the role of DHBTC in modulating inflammation and autophagy. In a lipopolysaccharide (LPS)-induced inflammatory model using RAW264.7, DHBTC exhibited potent anti-inflammatory activity. ELISA results (Supplementary Fig. 5a–f) showed that LPS stimulation significantly upregulated the expression of multiple key pro-inflammatory cytokines, including interleukin-1α (IL-1α), interleukin-1β (IL-1β), tumor necrosis factor-α (TNF-α), interleukin-8 (IL-8), interleukin-6 (IL-6), and prostaglandin E2 (PGE2). All treatment groups showed varying degrees of inhibition and the anti-inflammatory effect of DHBTC was the most significant, especially better than that of the BA and TA monomer groups.

**Fig. 6:**
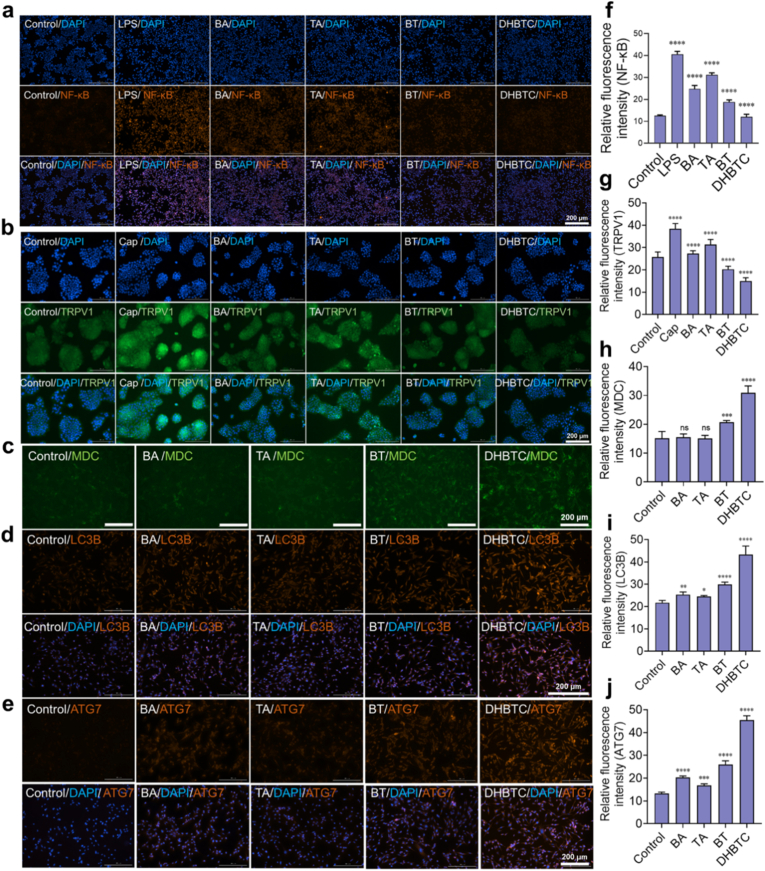
**Anti-inflammatory and Pro-autophagic Effects of DHBTC at the Cellular Level. a, f**, Immunofluorescence images and semi-quantitative analysis of the nuclear translocation of the NF-κB p65 subunit in LPS-stimulated RAW264.7 cells. Scale bar = 200 μm **b, g**, Immunofluorescence images and semi-quantitative analysis of TRPV1 expression in capsaicin (Cap) -stimulated cells. Scale bar = 200 μm. **c, h**, Monodansylcadaverine (MDC) staining showing autophagosome formation and its corresponding fluorescence intensity analysis. Scale bar = 200 μm **d, i**, LC3B immunofluorescence showing the formation of autophagic puncta and its quantitative analysis. Scale bar = 200 μm. **e, j**, Immunofluorescence images and semi-quantitative analysis of ATG7 protein expression. Scale bar = 200 μm. Results are shown as mean ± SD, ns = no significant difference, ∗p < 0.05, ∗∗p < 0.01, ∗∗∗p < 0.001, ∗∗∗∗p < 0.0001.

To trace the upstream molecular mechanism, we observed the subcellular localization of the core transcription factor of the inflammatory signaling pathway, the p65 subunit of nuclear factor-κB (NF-κB), using immunofluorescence staining. As shown in Fig. 6a, upon LPS stimulation, the p65 subunit rapidly translocated from the cytoplasm to the nucleus (orange fluorescence), thereby initiating the transcription of downstream inflammatory genes. However, in cells pretreated with DHBTC, the nuclear translocation of p65 was effectively blocked, with the majority of the p65 protein remaining in the cytoplasm. Semi-quantitative analysis confirmed that the inhibitory effect of DHBTC on p65 nuclear translocation was approximately 70.3 % of that observed in the LPS group, which was significantly higher than that observed in the BA and TA groups (Fig. 6f). We also examined the effects of DHBTC on the transient receptor potential vanilloid 1 (TRPV1) channel, which is closely associated with inflammation and sensory signal transduction. The expression of TRPV1 was significantly upregulated upon stimulation with the agonist capsaicin (Cap) and DHBTC again demonstrated the strongest inhibitory effect (−61 %) (Fig. 6b–g). Collectively, these results indicated that the anti-inflammatory action of DHBTC was achieved by inhibiting the key activation step of the NF-κB signaling pathway and modulating the TRPV1 channel.

Autophagy is a critical process for melanosome degradation BA has induces this process, therefore, we evaluated the effect of DHBTC on cellular autophagy. Using the autophagosome-specific fluorescent probe, monodansylcadaverine, we observed numerous bright green fluorescent puncta in DHBTC-treated cells, a typical sign of autophagosome formation, with significantly higher fluorescence intensity than that in the other groups (Fig. 6c–h). To further confirm the activation of autophagy, we performed immunofluorescence detection of a key protein marker of the autophagic process, microtubule-associated protein 1 light chain 3B (LC3B). In resting cells, LC3B is diffusely distributed in the cytoplasm, but upon induction of autophagy, it is converted to the lipidated LC3-II form and aggregates on the autophagosome membrane, forming distinct "puncta."

Semi-quantitative analysis showed that DHBTC significantly increased the number and density of intracellular LC3B-positive markers by approximately 1.9-fold compared with those in the control group, confirming enhanced autophagic flux (Fig. 6d–i). To further trace the upstream mechanism, we measured the expression of autophagy-related gene 7 (ATG7), a key E1-like activating enzyme essential for autophagosome formation. DHBTC was also able to most significantly upregulate the protein level of ATG7 (Fig. 6e–j). Collectively, DHBTC not only effectively inhibits inflammatory responses but also potently activates cellular autophagy. This dual synergistic action of "anti-inflammation" and "clearance" provides a solid cell-biological basis for its excellent therapeutic effects in vivo.

### 2.7 Topical application of DHBTC effectively ameliorates UVB-induced skin hyperpigmentation in mice

**Fig. 7:**
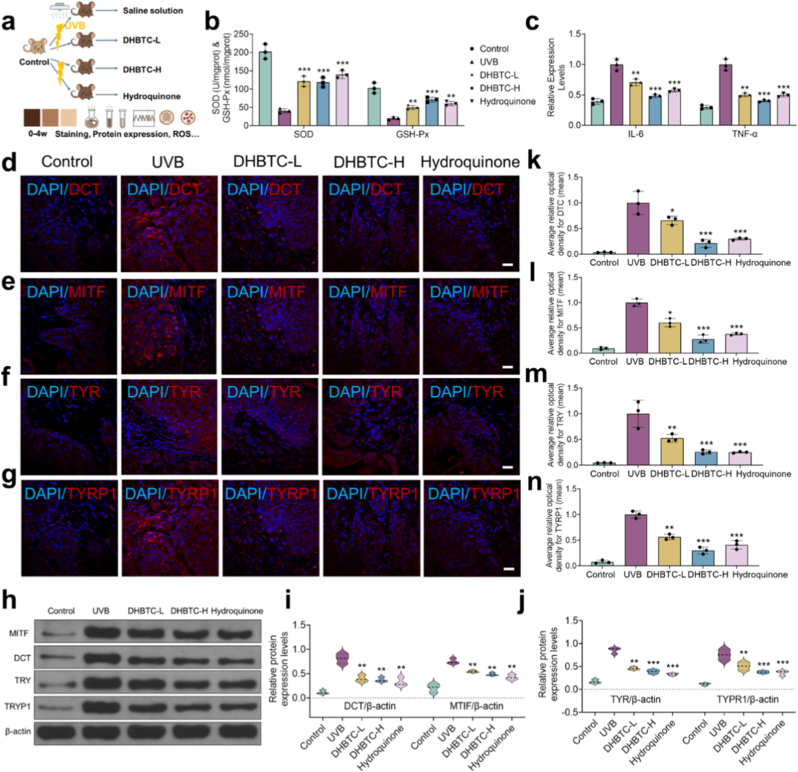
**In Vivo Therapeutic Efficacy of DHBTC in a UVB-Induced Mice Model. a**, Schematic diagram of the in vivo experimental design. **b**, Superoxide dismutase (SOD) and glutathione peroxidase (GSH-Px) activities in skin tissue homogenates. **c**, IL-6 and TNF-α levels in skin tissue homogenates. **d-g**, Immunofluorescence staining of DCT, MITF, TYR, and TYRP1 protein expression in skin tissue. Scale bar = 20 μm. **h-j,** Expression levels of different proteins in each treatment group. **k-n**, Semi-quantitative analysis of fluorescence intensity. Results are shown as mean ± SD, ns = no significant difference, ∗p < 0.05, ∗∗p < 0.01, ∗∗∗p < 0.001.

Based on the multifaceted biological activities observed in vitro, the in vivo therapeutic efficacy of DHBTC was validated in a UVB-induced C57BL/6 mouse model of skin hyperpigmentation (Fig. 7a). Mice were randomly divided into five groups: control (normal saline/time/day), model (UVB + normal saline/time/day), DHBTC-L (UVB + DHBTC: 2 mg/mL), DHBTC-H (UVB + DHBTC: 10 mg/mL), and hydroquinone cream (UVB + commercially 2 % available hydroquinone cream, 0.1g), for a total of 4 weeks.

First, throughout the entire experimental period, all mice in the experimental groups maintained normal appetite and were in good condition. No adverse reactions such as redness, swelling, scaling, or ulceration were observed in the skin. Based on this, we performed a series of biochemical and molecular biological tests on the skin tissue homogenate. ELISA results showed that the ROS level in the skin tissue was significantly decreased (−33.8 %) after DHBTC treatment compared with that in the model group (Supplementary Fig. 6a). The levels of the lipid peroxidation product, MDA, showed a similar trend (Supplementary Fig. 6b). Concurrently, the activities of the endogenous antioxidant enzymes, SOD and GSH-Px, were significantly restored and enhanced, respectively, after DHBTC-H treatment (Fig. 7b). In addition, the levels of the pro-inflammatory cytokines, IL-6 and TNF-α, were also significantly suppressed (Fig. 7c). These data indicate that the in vivo therapeutic effect of DHBTC is closely related to its potent antioxidant and anti-inflammatory capabilities, which is consistent with our in vitro results.

Next, we investigated the direct effects of DHBTC on melanogenesis. Biochemical analysis showed that DHBTC treatment significantly inhibited melanin synthesis in the skin tissue (Supplementary Fig. 6c). To further validate this inhibition at the protein level, we performed immunofluorescence analysis on skin tissue sections. In the model group, the expression levels of key proteins in the melanogenesis pathway, including dopachrome tautomerase (DCT), master transcription factor, MITF, tyrosinase (TYR), and tyrosinase-related protein-1 (TYRP1), were significantly upregulated. However, following DHBTC treatment, the expression of all these key proteins was significantly inhibited, with a substantial decrease in the fluorescence signal intensity (Fig. 7d–g). To corroborate these in situ findings with quantitative evidence, Western blot analysis was performed on skin tissue lysates. As shown in Fig. 7h–j, the protein abundance of MITF, TYR, and downstream enzymes was markedly downregulated in the DHBTC-treated groups compared to the model group, exhibiting a similar trend to the immunofluorescence results. Moreover, changes in these indicators showed a dose-dependent effect on DHBTC levels. Semi-quantitative analysis of the fluorescence intensity of each protein (Fig. 7k–n) and ELISA detection of TYR (Supplementary Fig. 6d) further confirmed this trend. These data collectively delineate a clear mechanism of action: DHBTC, through its multifaceted biological activities (antioxidant and anti-inflammatory), inhibits the upstream signals that activate MITF, thereby downregulating the expression of melanogenesis-related proteins and directly inhibiting the activity of key enzymes, ultimately leading to reduced melanin production.

### 2.8 Single-Cell Transcriptomics Reveals a mechanism of functional inhibition and compensatory transcriptional feedback

**Fig. 8:**
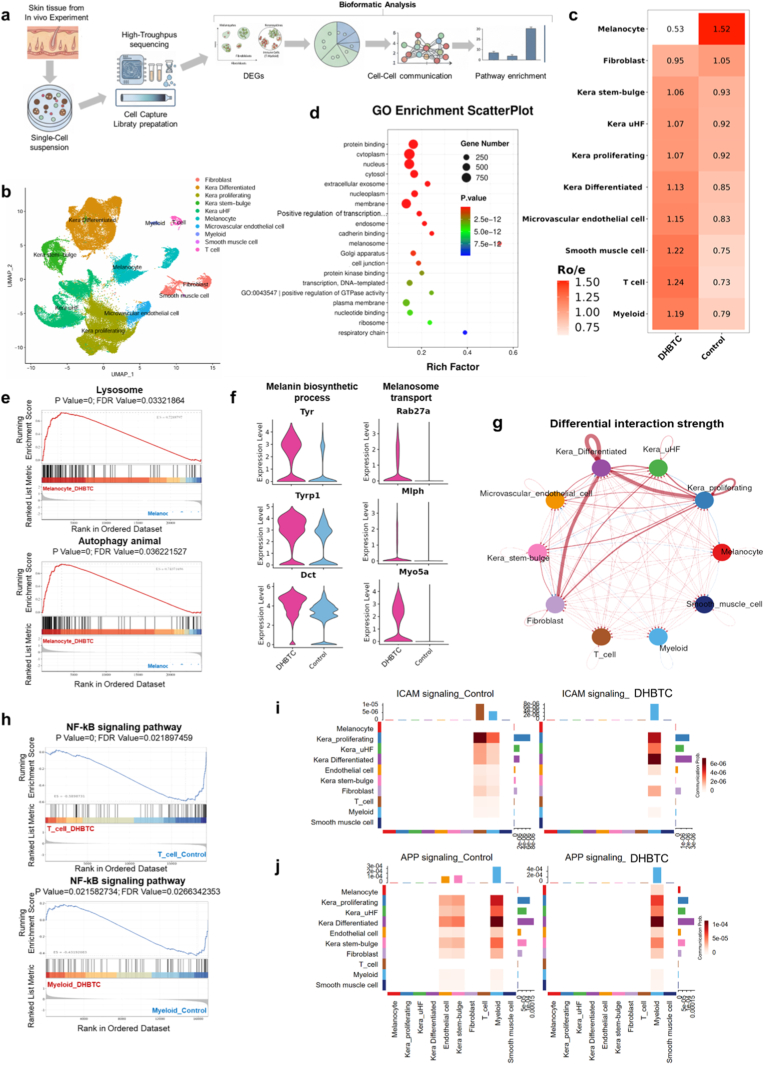
**Single-Cell Transcriptomics Reveals the Functional Inhibition and Compensatory Feedback Mechanism of DHBTC. a,** Schematic of the experimental workflow. **b**, UMAP plot of 89,860 cells from control and DHBTC-treated (BATA) skin samples, showing 10 major cell clusters. **c**, Ratio of observed to expected (Ro/e) plot illustrating the changes in cell type proportions before and after treatment. **d**, Gene Ontology (GO) enrichment analysis of differentially expressed genes between melanocytes from the DHBTC-treated and control groups. **e**, Violin plots showing the expression levels of key melanogenesis and transport genes in melanocytes from both groups. **f**, GSEA plots for the "lysosome" and "autophagy" pathways in melanocytes from the DHBTC-treated group. **g**, Circos plot of the cell-cell communication network in the skin microenvironment before and after treatment. **h**, Changes in communication strength in the pro-inflammatory ICAM and APP signaling pathways before and after treatment. **i**, Gene set enrichment analysis (GSEA) plots for the NF-κB signaling pathway in T and myeloid cells. Results are shown as mean ± SD, n = 3.

To dissect the molecular mechanisms by which DHBTC inhibits skin pigmentation, we performed single-cell RNA sequencing (scRNA-seq) on skin samples from the UVB model (Control) and DHBTC-L-treatment group (Fig. 8a). After filtering, the high-quality sequencing data yielded 89,860 cells. Through unbiased clustering analysis, we successfully constructed a cellular atlas of the skin tissue and identified 10 major cell clusters (Fig. 8b, Supplementary Fig. 7a). These clusters can be broadly classified into three categories: keratinocytes (comprising four subtypes: kera-differentiated, kera-proliferating, kera-stem-bulge, and Kera uHF), stromal cells (fibroblast, melanocyte, microvascular endothelial, and smooth muscle cells), and immune cells (T and myeloid cells).

**Fig. 9:**
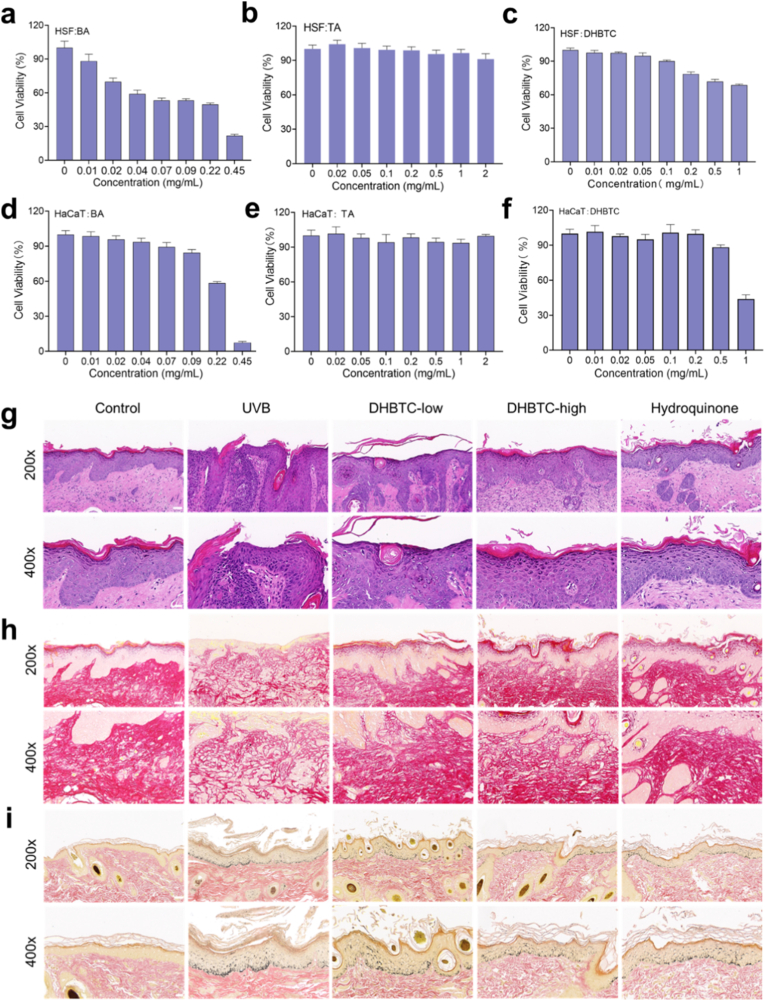
**Biosafety Assessment of the DHBTC Platform. a-c,** Cytotoxicity of Different Treatments (BA, TA and DHBTC) on HSF Cells. **d-f,** Cytotoxicity of Different Treatments (BA, TA and DHBTC) on HACAT Cells. **g-i,** Hematoxylin and eosin (H&E), Masson's, and Sirius Red stained images of skin tissue. (Upper: scale bar = 50 μm; Lower: scale bar = 20 μm).

To investigate the impact of DHBTC treatment on the overall cellular ecosystem of the skin, we performed a ratio of observed to expected (Ro/e) analysis on the cell types between the two groups. Significant remodeling of the skin microenvironment was observed after treatment. Consistent with the therapeutic goal, the relative proportion of melanocytes was significantly reduced after DHBTC treatment (Ro/e = 0.53). Conversely, the relative proportions of the two main immune cell populations, T (Ro/e = 1.24) and myeloid cells (Ro/e = 1.19), were significantly increased (Fig. 8c, Supplementary Fig. 7b–d). This shift in cellular composition suggests that DHBTC not only directly affects pigment cells, but may also exert its effects by modulating the immune microenvironment.

Next, we investigated the intrinsic transcriptional changes in melanocytes. Differential gene expression analysis between the two groups of melanocytes, followed by Gene Ontology (GO) enrichment analysis, revealed a contradiction of the macroscopic phenotype; in the DHBTC-treated group, the most highly and significantly enriched biological pathway was "melanosome" (Fig. 8d). To delve deeper into this paradoxical phenomenon, we examined the key genes within this pathway. Violin plots (Fig. 8e) clearly showed that in the melanocytes of the DHBTC-treated group, the transcript levels of the core enzyme genes responsible for the "Melanin biosynthetic process," including *Tyr, Tyrp1*, and *Dct*, were all significantly upregulated. Concurrently, the key complex genes responsible for "Melanosome transport," such as *Rab27a, Mlph*, and *Myo5a*, also showed consistent transcriptional upregulation. This systematic activation of the entire melanogenesis gene network contrasts with the significant whitening effect and protein level downregulation observed in our animal model (Fig. 7k).

We hypothesized that DHBTC potently inhibits melanin production and accumulation at the post-translational or functional levels, causing cells to sense a functional "melanin deficit, " thereby triggering a compensatory transcriptional feedback loop. The key to validating this hypothesis is to identify the downstream mechanisms of melanin clearance. Cellular autophagy is the primary mechanism for clearing organelles, such as melanosomes. To this end, we performed gene set enrichment analysis (GSEA), and the results (Fig. 8f) showed that in the melanocytes of the DHBTC-treated group, the lysosome- and autophagy-related pathways were both highly and significantly positively enriched. Furthermore, Western blotting and immunofluorescence experiments confirmed the enhancement of autophagy (Fig. 6c–e, h-**j, 7h**). To further functionally validate the mechanism predicted by scRNA-seq, we performed a phenotypic rescue experiment using the lysosomal inhibitor chloroquine (CQ). As expected, inhibition of lysosomal function significantly abrogated the DHBTC-induced degradation of Tyrosinase (TYR) protein (Supplementary Fig. S8a). Consistently, the addition of CQ effectively reversed the inhibitory effects of DHBTC on intracellular tyrosinase activity and melanin content, restoring them to near-control levels (Supplementary Fig. 8b and c). These data collectively demonstrate that DHBTC facilitates melanosome clearance by activating the autophagy-lysosomal system, which serves as the root cause triggering the upstream compensatory transcriptional feedback.

Finally, we investigated the biological significance of the increase in the proportion of immune cells following DHBTC treatment. Cell-cell communication analysis showed that after DHBTC treatment, the overall intercellular interaction network in the skin microenvironment was enhanced, with a more active signal exchange (Fig. 8g, Supplementary Fig. 9a–c). Analysis of the information flow also confirmed that the overall signal outflow intensity increased after DHBTC treatment (Supplementary Fig. 9d). However, an in-depth analysis of key inflammation-related pathways revealed the nature of this activity. In the control group, the ICAM (a marker of a strong inflammatory response) and APP signaling pathway (related to antigen presentation), centered on T and myeloid cells, both showed strong communication activity (Fig. 8h, Supplementary Fig. 9e). However, after DHBTC treatment, the overall communication strength between these two key pro-inflammatory pathways was significantly weakened. Analysis of specific ligand-receptor pairs within the signaling pathways also confirmed the attenuation of pro-inflammatory signals (Supplementary Fig. 9f). This indicates that the immune status of the skin microenvironment shifted from a pro-inflammatory to an anti-inflammatory or homeostatic state. To confirm this at the intracellular level, we performed GSEA on T and myeloid cells. The NF-κB signaling pathway, a key downstream transcription factor of the aforementioned inflammatory pathways, was significantly inhibited in both of these immune cell types (Fig. 8i). This finding is consistent with the cellular phenotype of inhibited NF-κB nuclear translocation observed in our immunofluorescence experiments (Fig. 6a–f).

In summary, the single-cell sequencing data profoundly reveal the dual mechanism of action of DHBTC: within melanocytes, it functionally inhibits pigmentation by inducing autophagy to clear melanosomes, which triggers a compensatory transcriptional feedback. Throughout the skin microenvironment, it modulates the immune status to a more quiescent, stable anti-inflammatory state by inhibiting key inflammatory pathways.

### 2.9 Comprehensive safety assessment of the DHBTC nanoplatform

To systematically evaluate the clinical application potential of the DHBTC nanoplatform, we conducted a multi-level safety assessment. First, we evaluated the cytotoxicity of DHBTC and its components on human keratinocytes (HaCaT), human skin fibroblasts (HSF), and murine monocyte-macrophages (RAW264.7) (Fig. 9a–f). The results revealed that free BA exhibited dose-dependent cytotoxicity in both HSF and HaCaT cells, although the two cell lines displayed distinct sensitivities (Fig. 9a–d). Specifically, for HSF cells, cell viability significantly declined when the concentration exceeded 0.01 mg/mL (Fig. 9a). In contrast, HaCaT cells exhibited higher tolerance, with a significant decrease in viability observed only at concentrations above 0.09 mg/mL (Fig. 9d). In comparison, DHBTC maintained the viability of HaCaT cells above 80 % even at concentrations as high as 0.5 mg/mL (Fig. 9f) and exhibited minimal cytotoxicity towards RAW264.7 (Supplementary Fig. 10a). TA alone also demonstrated a favorable safety profile (Supplementary Fig. 9b and e). These data indicate that our supramolecular hybridization strategy successfully mitigated the toxic effects of high-concentration BA, significantly broadening its safety window. This enhanced biocompatibility is attributed to the host-guest encapsulation by HP-β-CD, which likely prevents the acute osmotic shock and direct cell membrane perturbation often caused by high concentrations of free hydrophobic drugs. Furthermore, these results confirm that the biological activities observed in our previous experiments represent genuine pharmacological effects rather than artifacts resulting from cytotoxicity.

Next, to rule out permeation enhancement caused by physical damage, we analyzed the structural integrity of ex vivo porcine skin treated with DHBTC. H&E staining revealed that the stratum corneum remained dense and intact, with no signs of erosion or detachment (Supplementary Fig. 10c). Additionally, in situ optical monitoring via confocal Raman microscopy (Supplementary Fig. 10d) confirmed that the surface morphology of the DHBTC-treated group showed no significant differences compared to the saline control group. These results suggest that the enhanced permeation of DHBTC is driven by a thermodynamic solubility gradient rather than by chemical disruption of the skin barrier.

Finally, we validated the long-term safety in a UVB-induced pigmentation mouse model over a 4-week period. Throughout the experiment, the mice exhibited normal diet, activity levels, and weight gain. H&E staining of skin sections revealed that UVB irradiation led to significant epidermal thickening and inflammatory cell infiltration in the superficial dermis of the model group. DHBTC treatment, especially at a high dose, effectively reversed these pathological changes, restored epidermal thickness to a normal level, and significantly alleviated the inflammatory response (Fig. 9g). Masson's trichrome staining further revealed the extent of pigment deposition; many dense melanin granules were present in the basal layer of the epidermis in the model group (Fig. 9h). In the DHBTC-treated group, the number and density of melanin granules were significantly reduced and pigment distribution within the epidermis returned to near-normal levels. Furthermore, Sirius Red staining and semi-quantitative results indicated that UVB irradiation caused the disorganization, fragmentation, and degradation of collagen fibers in the dermis (Fig. 9i, Supplementary Fig. 10b). However, DHBTC effectively protects the collagen network and maintains its dense and orderly structure, which not only protects the skin but also demonstrates its anti-photoaging effect in vivo. Moreover, skin sections from the administration site showed no evidence of erythema, edema, or allergic reactions.

In summary, these results establish the superior biosafety of DHBTC, confirming that it achieves efficient therapeutic effects without sacrificing biocompatibility or tissue integrity, thereby laying a solid foundation for its future clinical application.

## 3 Discussion and conclusion

A central finding of this study was the paradoxical observation revealed by single-cell transcriptomics: our nanoplatform potently reversed hyperpigmentation while systematically upregulating the entire melanogenesis gene network in melanocytes. We propose that this reflects a sophisticated mechanism of "functional inhibition," where the therapeutic effect is achieved by disrupting post-translational processes rather than suppressing gene expression.

### 3.1 Autophagy-mediated functional inhibition and compensatory

Our data suggests that DHBTC induces functional inhibition through a specific "autophagy-dependent clearance" mechanism. The efficient delivery of BA triggers potent activation of the autophagy-lysosome pathway, leading to the rapid degradation of melanosomes (melanophagy). This creates a functional "melanin deficit" within the cell. The cellular homeostatic machinery senses this deficit and responds by activating MITF, triggering a "futile" compensatory transcriptional upregulation of melanogenesis genes (*Tyr, Tyrp1, Dct*). However, this compensation fails to restore pigment levels because the synthesized proteins are continuously targeted for degradation. Additionally, the upregulation of *Hsp90* transcripts likely reflects cellular stress that may compromise chaperone function, further destabilizing client proteins like tyrosinase. This synthesis-degradation futile cycle explains the discrepancy between high mRNA levels and low protein/melanin levels.

### 3.2 Modulation of inflammation and remodeling of the immune

Distinct from the direct clearance effect on melanocytes, DHBTC operates on a second dimension by remodeling the cutaneous immune microenvironment. Single-cell analysis and biological assays demonstrated that DHBTC shifts the skin from a pro-inflammatory to a homeostatic state. Mechanistically, this is achieved by inhibiting the NF-κB signaling pathway and modulating TRPV1 channels. The intercellular communication network analysis revealed that DHBTC significantly attenuated the pro-inflammatory signaling intensity between immune cells and skin resident cells. This dual-action strategy-directly disabling pigment accumulation via autophagy while simultaneously resolving the underlying inflammatory triggers-accounts for the robust and durable therapeutic effects observed in vivo.

### 3.3 Assessment of the AI model: advantages, limitations, and generalizability

The primary advantage of our AI-assisted strategy lies in its unique dual-targeting capability. Unlike traditional computational screening approaches that focus solely on structural compatibility, such as binding energy metrics, our hybrid RF-BP model integrates "Tyrosinase Inhibitory Potential" as a core input feature. This design ensures that the identified co-formers, such as Tranexamic Acid, act not merely as inert solubilizers but as synergistic bioactive agents. This transition from "inert carriers" to "functional carriers" represents a significant leap in formulation design logic, allowing for the simultaneous optimization of physicochemical properties and therapeutic efficacy.

Despite the promising accuracy achieved on the test set, we acknowledge the limitations inherent to our data-driven approach. The model was trained on a dataset of 159 high-fidelity experimental entries, which serves as a small but precise foundation sufficient to cover the specific chemical space of hydrophobic flavonoids and hydrophilic amino acid or acid ligands. However, this imposes a chemical space constraint; the model's predictive accuracy may decrease when applied to structurally distinct compounds, such as macromolecules or steroids, without prior re-training. Furthermore, although we employed feature reduction via Random Forest and strict train/test splitting to mitigate overfitting, the relatively small sample size precludes the use of complex deep learning architectures that require massive data ingestion.

Regarding generalizability, it is crucial to distinguish between the specific model parameters and the broader design methodology. While the current weight matrix is optimized specifically for the baicalin-tranexamic acid system, the proposed "Inverse Design Workflow" (comprising small-sample library construction, RF feature reduction, BP prediction, and genetic algorithm optimization) constitutes a universal framework. This computational pipeline is highly transferable and can be readily adapted to solve carrier screening challenges for other BCS Class II/IV drugs by retraining with domain-specific data. Future work will focus on expanding the dataset through automated high-throughput experimentation to further broaden the model's applicability and robustness.

## 4 Experimental section

### 4.1 Materials

Baicalin (BA, purity ≥98 %) and Tranexamic Acid (TA, purity ≥99 %) were purchased from Shanghai Yuanye Bio-Technology Co., Ltd. (Shanghai, China). Hydroxypropyl-β-cyclodextrin (HP-β-CD, degree of substitution 4.2–5.1) was purchased from Sigma-Aldrich (St. Louis, MO, USA). All solvents used for High-Performance Liquid Chromatography (HPLC) analysis were of chromatographic grade, and all other chemical reagents were of analytical grade. Human keratinocytes (HaCaT), Human Skin Fibroblasts (HSFs), and murine macrophages (RAW264.7) were obtained from the Cell Bank of the Chinese Academy of Sciences (Shanghai, China). Dulbecco's Modified Eagle Medium (DMEM), fetal bovine serum (FBS), penicillin-streptomycin solution, and trypsin-EDTA solution were purchased from Gibco (Grand Island, NY, USA). Antibodies against DCT, MITF, and TYR were purchased from Merck (Darmstadt, Germany). The Cell Counting Kit-8 (CCK-8), DCFH-DA reactive oxygen species (ROS) assay kit, superoxide dismutase (SOD), catalase (CAT), glutathione peroxidase (GSH-Px), and malondialdehyde (MDA) activity assay kits, as well as all secondary antibodies and anti-fade mounting medium, were purchased from Beyotime Biotechnology (Shanghai, China).

### 4.2 Preparation and characterization of nanoplatforms

#### 4.2.1 Preparation of the BT assembly

Baicalin and tranexamic acid were accurately weighed according to various molar ratios (1:1, 1:2, 1:3, 2:1, and 3:1). The two solid powders were co-dissolved in 20 mL of methanol in a 50 mL round-bottom flask to ensure complete dissolution, forming a clear, pale-yellow solution. The solution was then continuously stirred at 500 rpm for 8 h at room temperature (25 °C) under dark conditions. Following the reaction, the methanol was slowly removed by rotary evaporation under reduced pressure (<50 mbar) in a 40 °C water bath until a uniform solid film formed on the inner wall of the flask. To ensure complete removal of residual solvent, the resulting solid was carefully collected with a spatula and dried in a vacuum oven at 50 °C and −0.1 MPa for 48 h. The final product, a pale-yellow, loose BT assembly powder, was stored in a desiccator until further use.

#### 4.2.2 Construction of the DHBTC

The pre-prepared BT assembly powder and hydroxypropyl-β-cyclodextrin (HP-β-CD) were accurately weighed according to specified ratios and co-added to 20 mL of high-purity deionized water (Milli-Q grade). The mixture was sonicated for 30 min in a bath sonicator (40 kHz, 100 W) under an ice-water bath to facilitate dispersion. The resulting uniform, milky-white suspension was then transferred to a magnetic stirrer and stirred continuously at 500 rpm for 24 h at room temperature (25 °C) under dark conditions. After the reaction, the solution was transferred to a lyophilization flask and pre-frozen at −80 °C for at least 12 h. Subsequently, the frozen sample was transferred to a freeze-dryer (Labconco FreeZone) and lyophilized under high vacuum (<10 Pa) at a condenser temperature of < −50 °C for 48 h. The final product, a pale-yellow DHBTC powder, was stored in a desiccator.

#### 4.2.3 Physicochemical characterization

Nuclear Magnetic Resonance (NMR), Fourier-Transform Infrared (FTIR), and Raman spectroscopy analyses were performed using a Bruker 400 MHz NMR spectrometer, a Thermo Nicolet 380 FTIR spectrometer, and a Horiba LabRAM HR Evolution Raman microspectrometer, respectively. Thermogravimetric Analysis (TGA) and Differential Scanning Calorimetry (DSC) were conducted using a Mettler TGA2 thermogravimetric analyzer and a Mettler DSC3 differential scanning calorimeter, respectively. Approximately 10 mg of each sample was heated at a scanning rate of 5 °C min^−1^. Viscosity, conductivity, and pH measurements were performed using an NDJ9S digital viscometer (Lichen, Shanghai), a DDS-307 digital conductivity meter (INESA, Shanghai), and a Mettler FE28 pH meter, respectively.

### 4.3 In vitro transdermal experiments

New porcine skin of Bama miniature pigs (male, aged 30 days, weight of 2.5–3.5 kg, obtained from Taihe Biotechnology), with a thickness of about 500 μm, was placed between the donor and receiver cells of the TP-6 Franz diffusion equipment (Tianjin Jingtuo). The stratum corneum of the porcine skin was settled upward with 2 mL of the sample solution in the donor cell, and the receiver cell was filled with PBS. Under continuous stirring of 300 rpm min^−1^ at 32 °C, an aliquot (1 mL) of the receiver solution was collected every 2 h and replaced by fresh PBS solution (1 mL). The BA concentration in the receiver solution was determined using an Agilent 1260 Infinity II LC system, and the cumulative penetration per unit area of HP9 (*Q*_s_, μg cm^−2^) was calculated according to Equation (1).

(1) $$Q_{s}=C_{\text{sn}}\times \frac{V_{s}}{A_{s}}+\sum_{i=1}^{n‐1}C_{\text{si}}\times \frac{S}{A_{s}}$$

where *C*_si_ and *C*_sn_ are the BA concentrations in the ith and nth sampling solutions obtained from the receiver cell, respectively, while *V*_s_, *A*_s_, and *S* are the receiver volume, the effective diffusion area, and the solution volume per sampling from the receiver cell, respectively.

### 4.4 Cell experiments

#### 4.4.1 Cytotoxicity

In the logarithmic phase, HaCaT, HSF, and Raw264.7 cells were cultured in 96-well plates (1 × 10^4^ cells per well) with DMEM medium overnight. Subsequently, the medium was replaced with fresh DMEM containing samples at different concentrations. After a subsequent incubation for 48 h, CCK-8 solution (10 μL per well) was added and incubation was continued for a further 1 h. Thereafter, the optical density (OD) at 450 nm was recorded using a BioTek 800 TS microplate reader, and the cell viability was calculated according to Equation (2):

(2) $$Cell\;viability\;\left(\%\right)=\frac{A_{\text{sample}}-A_{\text{blank}}}{A_{\text{control}}-A_{\text{blank}}}$$

where *A*_blank_, *A*_control_, and *A*_sample_ are the ODs of the wells with no cells or treatment compounds, cells with no treatment compounds, and cells with treatment compounds, respectively. The IC_10_ and IC_50_ values are defined as the concentrations that led to 10 % and 50 % cell inhibition, respectively.

#### 4.4.2 Wound healing assay

The migratory ability of HSFs was assessed using a scratch assay. HSFs were seeded into 6-well plates and grown to full confluence. A sterile pipette tip was used to create a linear scratch across the cell monolayer. The cells were then washed to remove debris and incubated with serum-free medium containing different test samples. Images of the scratch were captured at 0 and 24 h using an inverted microscope. The rate of wound healing was quantified by measuring the scratch area using ImageJ software.

#### 4.4.3 Reactive oxygen species assay

Intracellular ROS levels were measured using the fluorescent probe DCFH-DA. Cells were seeded in confocal dishes. After treatment with UVA or H_2_O_2_ in the presence or absence of the test samples, the cells were incubated with the DCFH-DA probe for 30 min. The fluorescence was then observed using a fluorescence microscope or quantified using a microplate reader.

#### 4.4.4 Antioxidant enzyme and lipid peroxidation assays

The activities of endogenous antioxidant enzymes and the level of lipid peroxidation were determined using commercial assay kits. Following treatment, cells were collected and lysed. The activities of SOD, CAT, and GSH-Px, as well as the concentration of MDA, were measured in the cell lysates according to the manufacturer's protocols.

#### 4.4.5 Immunofluorescence staining

Following treatment, cells were fixed with 4 % paraformaldehyde, permeabilized with 0.2 % Triton X-100, and blocked with bovine serum albumin (BSA). The cells were then incubated with primary antibodies against NF-κB p65, TRPV1, LC3B, or ATG7 at 4 °C overnight. After washing, the cells were incubated with fluorescently labeled secondary antibodies for 1 h at room temperature. Nuclei were counterstained with DAPI. The stained cells were then visualized using a confocal laser scanning microscope (CLSM).

#### 4.4.6 Western blot analysis

Total protein was extracted from the HaCat cells, and the concentration was determined using a BCA protein assay kit. Equal amounts of protein were separated by SDS-PAGE and transferred to a polyvinylidene fluoride (PVDF) membrane. The membrane was blocked with 5 % non-fat milk and then incubated with primary antibodies against Nrf2, NQO1, or β-actin at 4 °C overnight. After washing, the membrane was incubated with HRP-conjugated secondary antibodies for 1 h at room temperature. The protein bands were visualized using an ECL chemiluminescence kit and imaged with a gel imaging system.

### 4.5 Animal experiments

All animal experiments were approved by the Institutional Animal Care and Use Committee of our university. Six-to eight-week-old C57BL/6 mice were used for the study. A hyperpigmentation model was established by depilating the dorsal skin of the mice and irradiating it with a UVB lamp (280–320 nm) at a dose of 50 mJ/cm^2^, three times per week for four weeks. The mice were randomly divided into four groups: a model group, a low-dose DHBTC group, a high-dose DHBTC group, and a positive control group (hydroquinone cream). The respective formulations were applied topically once daily. At the end of the experiment, the following procedures were performed: **a**, Dorsal skin samples were collected, fixed in 4 % paraformaldehyde, embedded in paraffin, and sectioned. The sections were then subjected to Hematoxylin and Eosin (H&E), Masson's trichrome, and Sirius Red staining and observed under a light microscope. **b**, Skin tissue was homogenized, and the levels of ROS, MDA, IL-6, and TNF-α, as well as the activities of SOD, GSH-Px, and tyrosinase, were measured using the corresponding commercial assay kits. **c**, Frozen sections of skin tissue were prepared and stained with primary antibodies against DCT, MITF, TYR, and TYRP1, following the same procedure as for cellular immunofluorescence, and were subsequently observed.

### 4.6 Single-cell RNA sequencing and bioinformatic analysis

Skin tissues from the model group and the high-dose DHBTC group were digested with collagenase and dispase to prepare single-cell suspensions. Single-cell capture and library construction were performed using the 10x Genomics Chromium platform, and sequencing was carried out on an Illumina Nova Seq 6000. The raw sequencing data were aligned and quantified using the Cell Ranger software suite. Subsequent analysis was performed using the Seurat package in R. The cells were subjected to quality control filtering, normalization, and dimensionality reduction (Principal Component Analysis, PCA), followed by clustering and visualization using Uniform Manifold Approximation and Projection (UMAP). Cell clusters were annotated based on known marker genes. Differentially expressed genes were identified using the FindMarkers function. Gene Ontology (GO) enrichment analysis was performed using the clusterProfiler package, and Gene Set Enrichment Analysis (GSEA) was conducted using the GSEA Base package. Intercellular communication network analysis was performed using the Cell Chat package.

### 4.7 AI model construction and ligand screening

A predictive model was developed from a dataset of 159 experimental entries. Key molecular descriptors, including SMILES, number of hydrogen bond donors (HBD), molecular weight, tyrosinase inhibitory potential, and topological polar surface area (TPSA), were extracted using RDKit and selected as input features via a random forest algorithm. A back-propagation (BP) neural network was constructed and trained on this dataset (120 training, 39 testing entries). The validated model was then integrated with a genetic algorithm to perform a global optimization search under predefined physicochemical and biological constraints. The genetic algorithm was configured with optimized parameters for crossover, mutation, and population size over 100 iterations to identify the optimal ligand.

### 4.8 DFT calculations

The structures of BT and DHBTC were optimized at the B3LYP/6-31G∗ level of theory, and their single-point energies were subsequently calculated at the wB97M-V/ma-def2-TZVP level using the ORCA 5.0.4 program. Electrostatic potential (ESP), atoms-in-molecules (AIM), and Independent Gradient Model based on Hirshfeld partition (IGMH) analyses were performed using the Multiwfn 3.8 dev and VMD 1.9.3 software packages.

### 4.9 MD simulation of transdermal delivery

MD simulations were performed using GROMACS 2021.5, with the methods as previously described. The topology parameters for BT, ceramide (CER), cholesterol (CHL), and free fatty acids (FFA) were determined using the CHARMM36m force field. Using the membrane builder module in CHARMM-GUI, the system containing the skin barrier complex was placed in a cubic box of 8 × 8 × 25 nm. Two solvent environments, pure water and a 0.3 M BT solution, were established using the TIP3P water model. Energy minimization was performed to eliminate unreasonable intramolecular contacts within the system. Following pre-equilibration, the transdermal transport of HP9 was simulated using umbrella sampling under NPT (constant particle number, pressure, and temperature) conditions at 310 K and 1 bar.

### 4.10 Statistical analysis

All results were analyzed using GraphPad Prism 9.5 software and are expressed as the mean ± SD. The paired *t*-test was used to compare clinical data, whereas one-way ANOVA was used to compare all other results. Statistical significance was set at p < 0.05.

## CRediT authorship contribution statement

**Tianqi Liu:** Writing – original draft, Visualization, Investigation, Formal analysis, Conceptualization. **Liang Chen:** Investigation, Funding acquisition, Formal analysis, Data curation. **Xiaoyu Zhao:** Software, Resources, Methodology, Investigation, Formal analysis. **Min Xie:** Writing – review & editing, Investigation, Funding acquisition, Formal analysis. **Ling Xie:** Writing – review & editing, Supervision, Investigation, Funding acquisition, Formal analysis. **Mi Wang:** Writing – review & editing, Supervision, Investigation, Formal analysis. **Zhenyuan Wang:** Writing – review & editing, Resources, Investigation, Formal analysis, Data curation. **Jiaheng Zhang:** Writing – review & editing, Supervision, Investigation, Funding acquisition, Formal analysis, Data curation, Conceptualization.

## Ethics approval and consent to participate

C57BL/6J mice purchased from three Gorges University Animal Centre (Yichang, Hubei, China) was used in this study. The license number is SYXK (er) 2022-0012 (202510187). All animal experiments were performed in accordance with animal research protocols approved by the Animal Care and Use Committee of Peking University. In the in vivo experiments, mice were shaved (Back: 2 × 3 cm^2^) on the 50th day from birth and the depilated areas were coated with saline, 5 % minoxidil, CaT containing 2 % copper peptides, and CaT-ME containing 2 % copper peptides daily after shaving, with 6 mice per group. Application twice daily for 21 days, followed by fasting for 12 h and then execution of the mice for sampling. Temperature was measured daily before and after administration to evaluate the body temperature changes of the mice. Measurement time 20 min before dosing, 0 min, 20 min, 60 min, 120 min after dosing. The body weight of the mice was measured at specific times. Temporal profiles of hair phenotypic transformation were obtained through real-time observation of hair regeneration in the mice. Fixed size skin pieces were peeled and weighed, and weighed again after hair removal, and three pieces were taken for each group for repeated measurements to obtain the weight of newly grown hairs in the mice.

## Declaration of competing interest

The authors declare the following personal relationships which may be considered as potential competing interests: Liang Chen, Min Xie, and Ling Xie are currently employed by Shanghai Le-Surely Biotechnology Co., Ltd and Shanghai Chuanmei Industrial Co., Ltd. Zhenyuan Wang and Jiaheng Zhang are currently employed by Shenzhen Shinehigh Innovation Technology Co., Ltd. and Gd Shinesky Medical Supplies Health Technology Co., Ltd.

## Appendix A Supplementary data

The PDF file includes:Figs. S1–S9. Other Supplementary Material for this manuscript includes the following: TableS1,S2.
