# Supplementary material for: An AI-assisted designed supramolecularly engineered nanoplatform reverses pigmentation by triggering an ineffective compensatory melanin production program
Source: Bioact Mater. 2026 Jan 24;60:243–60. doi: 10.1016/j.bioactmat.2026.01.027 (PMC12860373; doi:10.1016/j.bioactmat.2026.01.027)
Supplement: Multimedia component 1 [file mmc1.docx]

Supporting Information

**An AI-assisted designed supramolecularly engineered nanoplatform reverses pigmentation by triggering an ineffective compensatory melanin production program**


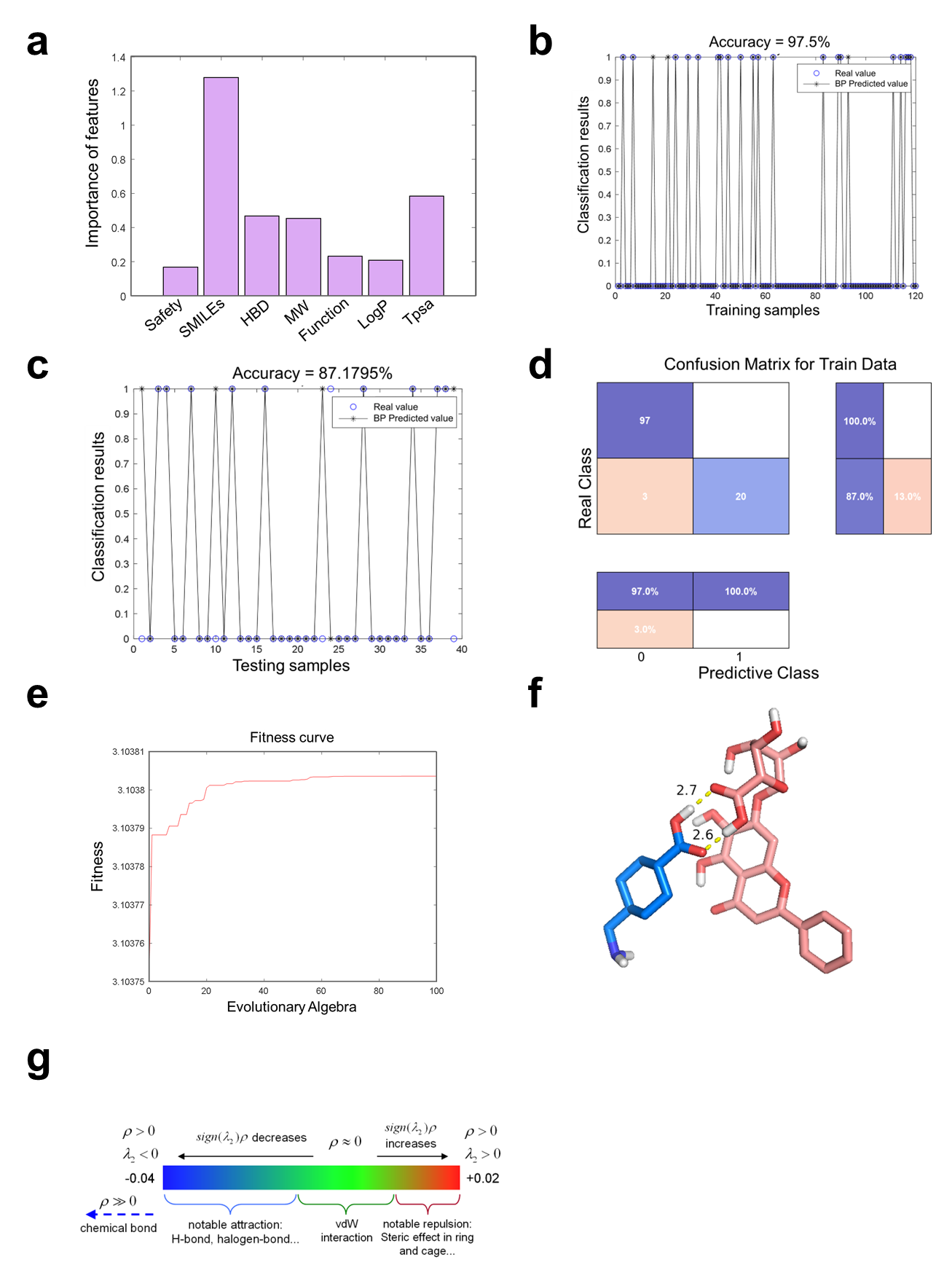


**Figure S1. AI-Driven Design, Theoretical Validation, and Hierarchical Self-Assembly Strategy of DHBTC.** **a**,Feature importance ranking graph. **b**, Classification results of the training dataset. **c**, Classification results of the test dataset. **d**, Confusion matrix of the training dataset. **e**, Algorithm iteration performance curve

**
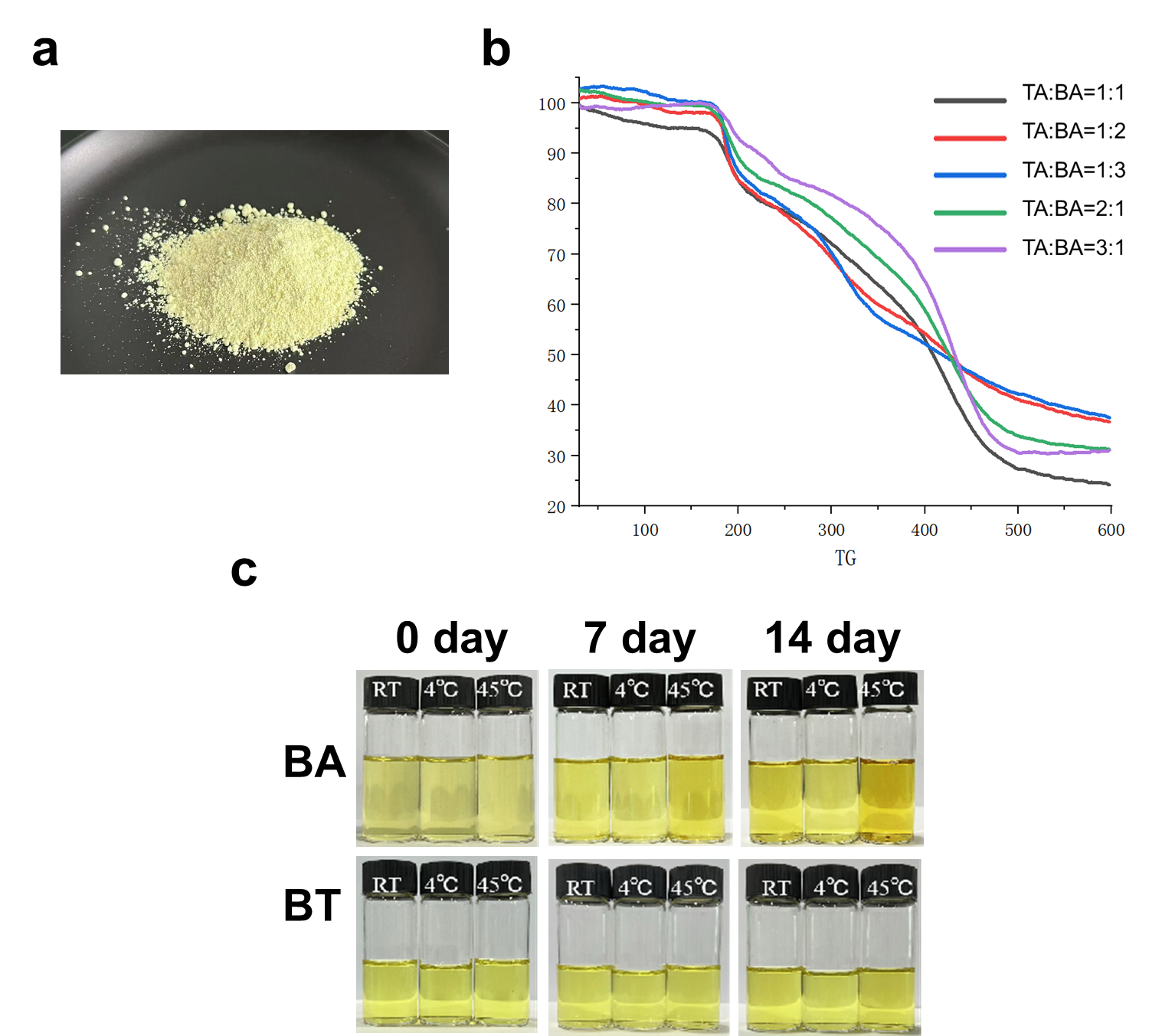
**

**Figure S2. Preparation, Characterization, and Performance Analysis of the BT Supramolecular Assembly. a**, Photo of BT. **b**, Thermogravimetric curves of BT at different molar ratios. **c**, Macroscopic images of BA and BT at different times and temperatures.


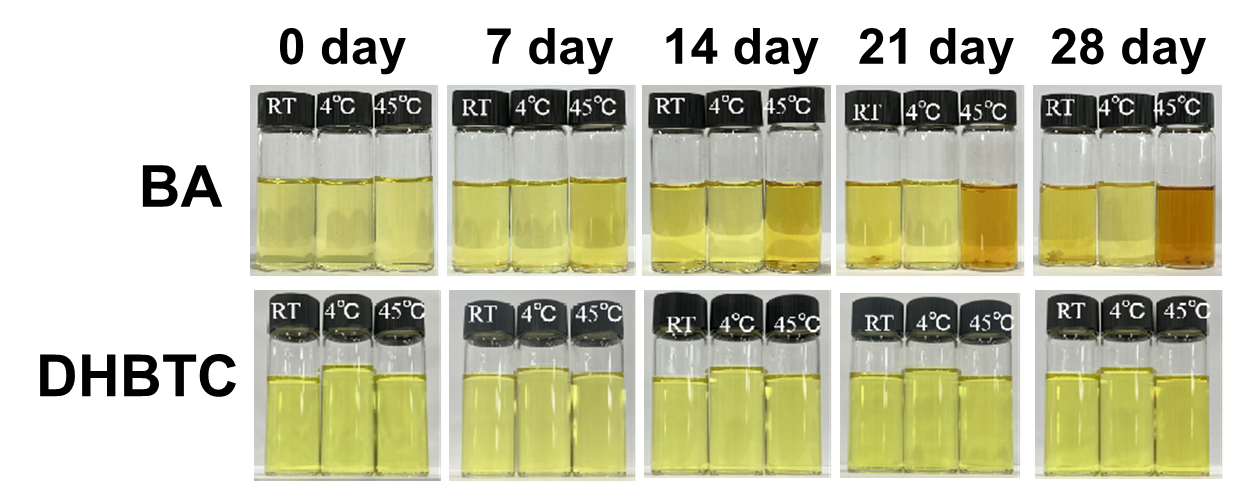


**Figure S3.** Macroscopic images of BA and DHBTC at different times and temperatures.


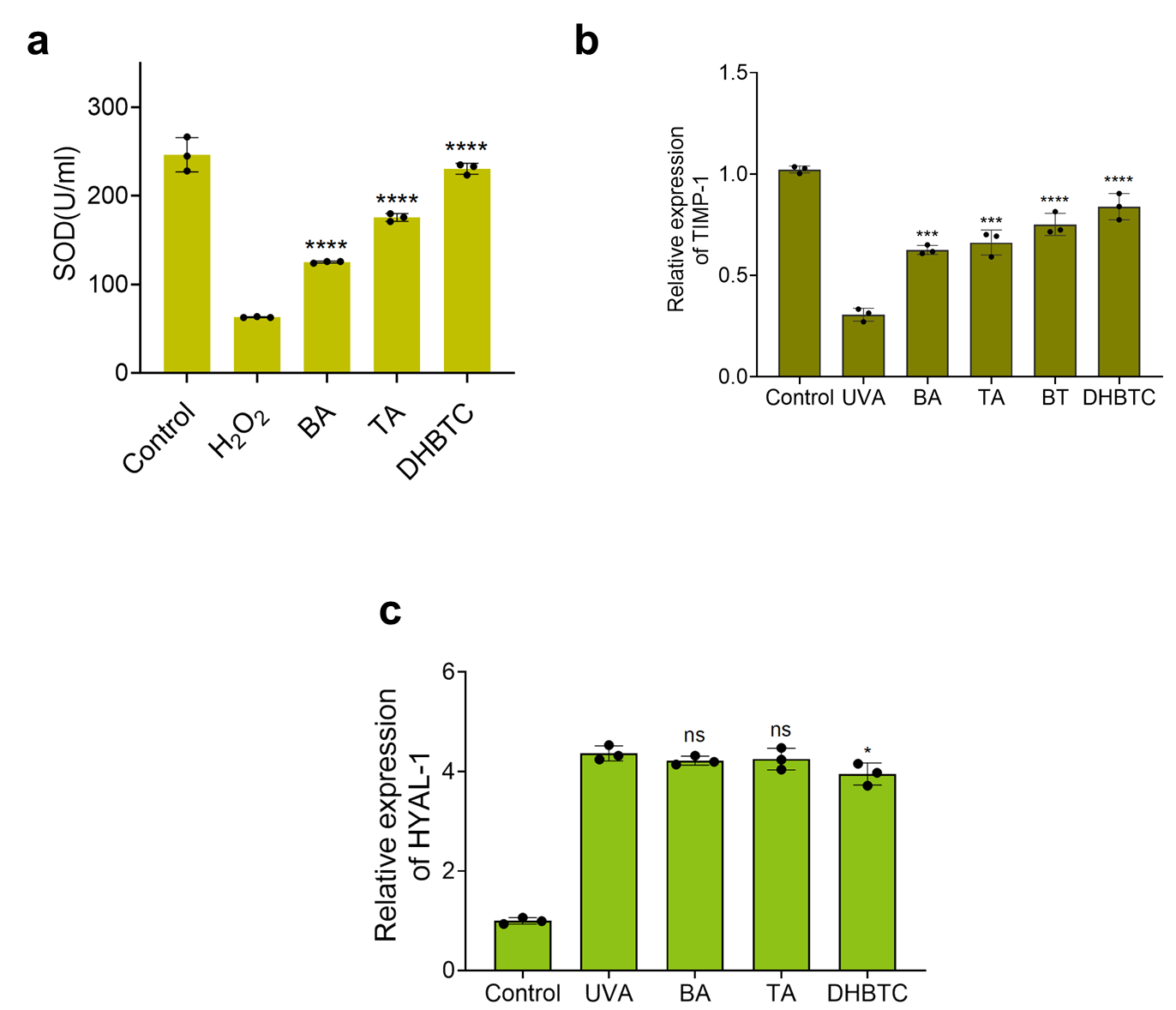


**Figure S4.** **Antioxidant, and Anti-Photoaging Functions of DHBTC**. **a**, SOD expression was measured by ELISA. **b, c**, TIMP-1 and HYAL-1 expression was measured by QPCR. Results are shown as the mean ± SD for n = 3; ns: no statistical difference,*p <0.05, **p <0.01, ***p <0.001, ****p <0.0001.

**
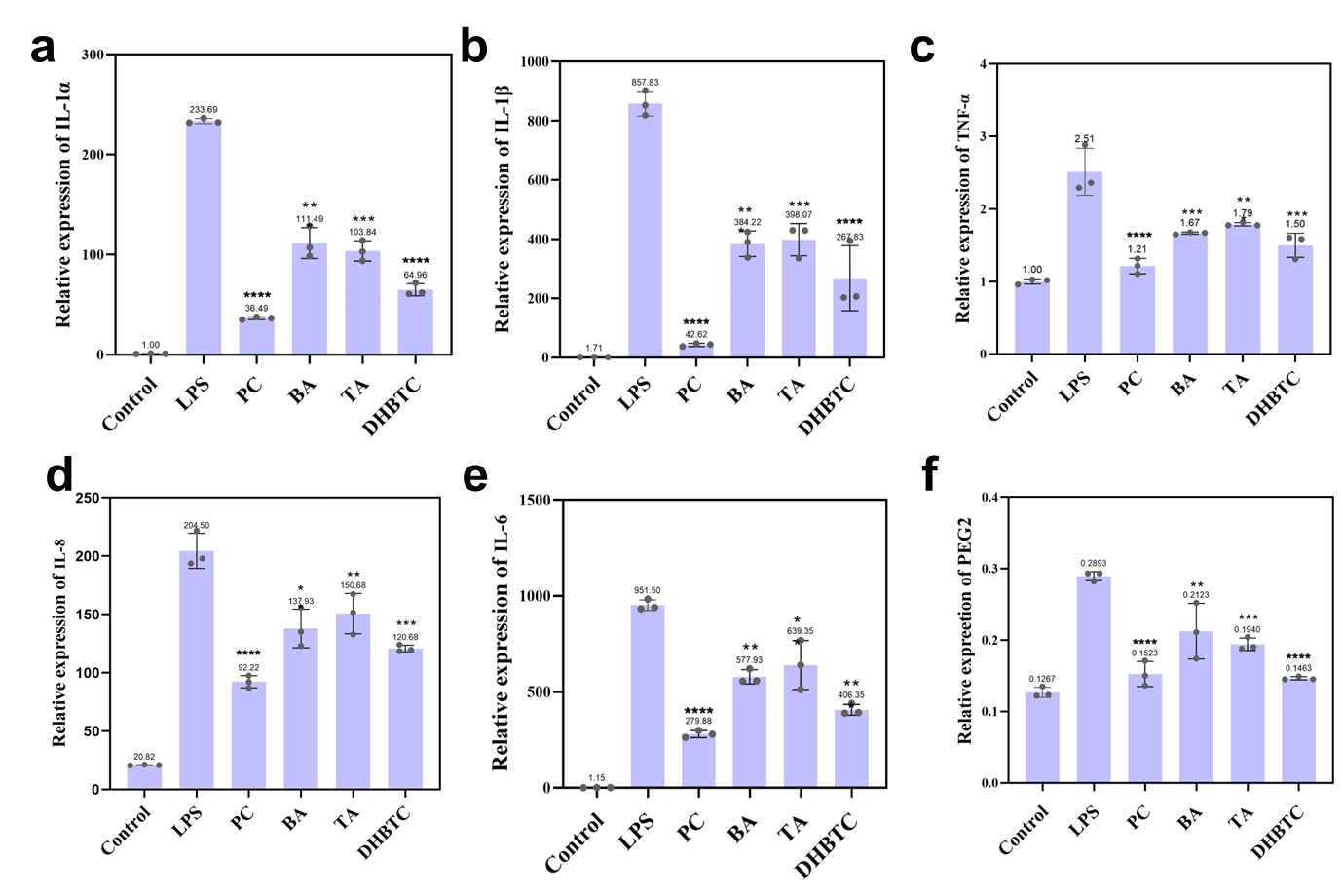
**

**Figure S5.** Expression of IL-1α, IL-1β, TNF-α, IL-8, IL-6, and PEG2 in Different Treatment Groups. Results are shown as the mean ± SD for n = 3; ns: no statistical difference,*p <0.05, **p <0.01, ***p <0.001, ****p <0.0001.


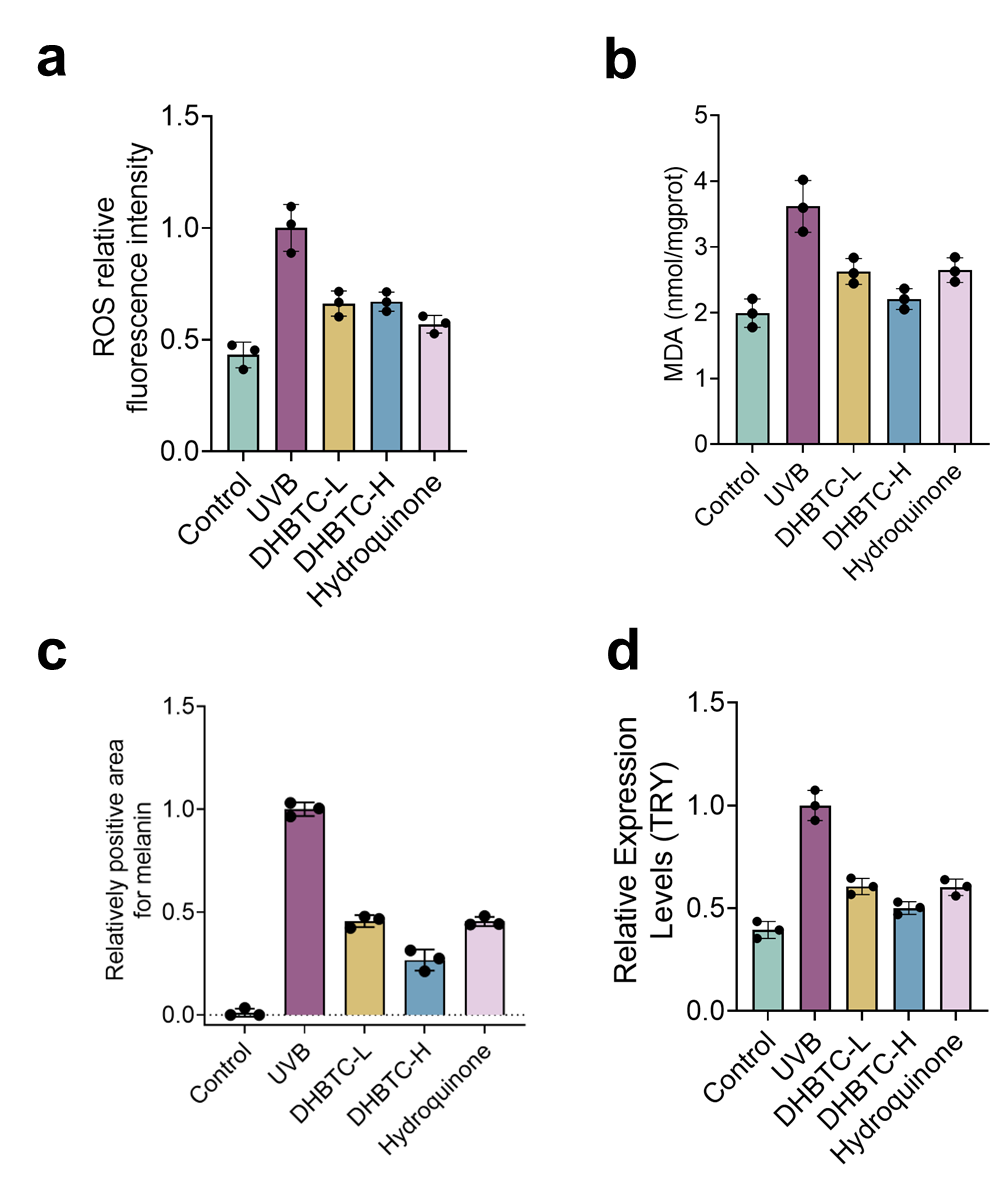


**Figure S6.** **a**, ROS expression in different treatment groups. **b**, MDA expression in different treatment groups. **c**, Relative quantitative analysis of melanin in different treatment groups. **d**, Quantitative analysis of TRY.

**
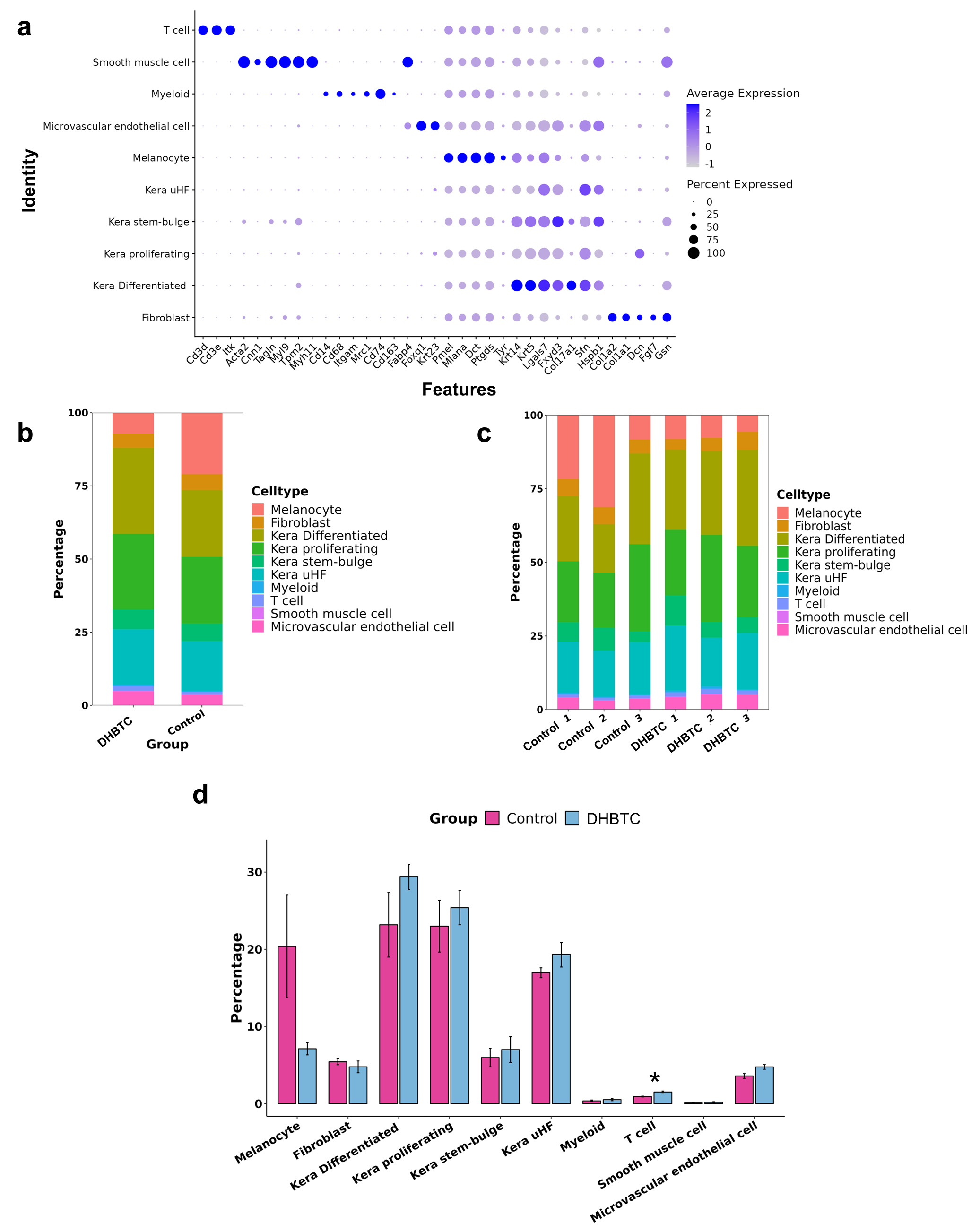
**

**Figure S7.** **a**. Dot plot of gene expression in cells. **b,c**. Cell proportions in groups (b) and samples (c). **d**. Histogram of the percentages of each cell type.


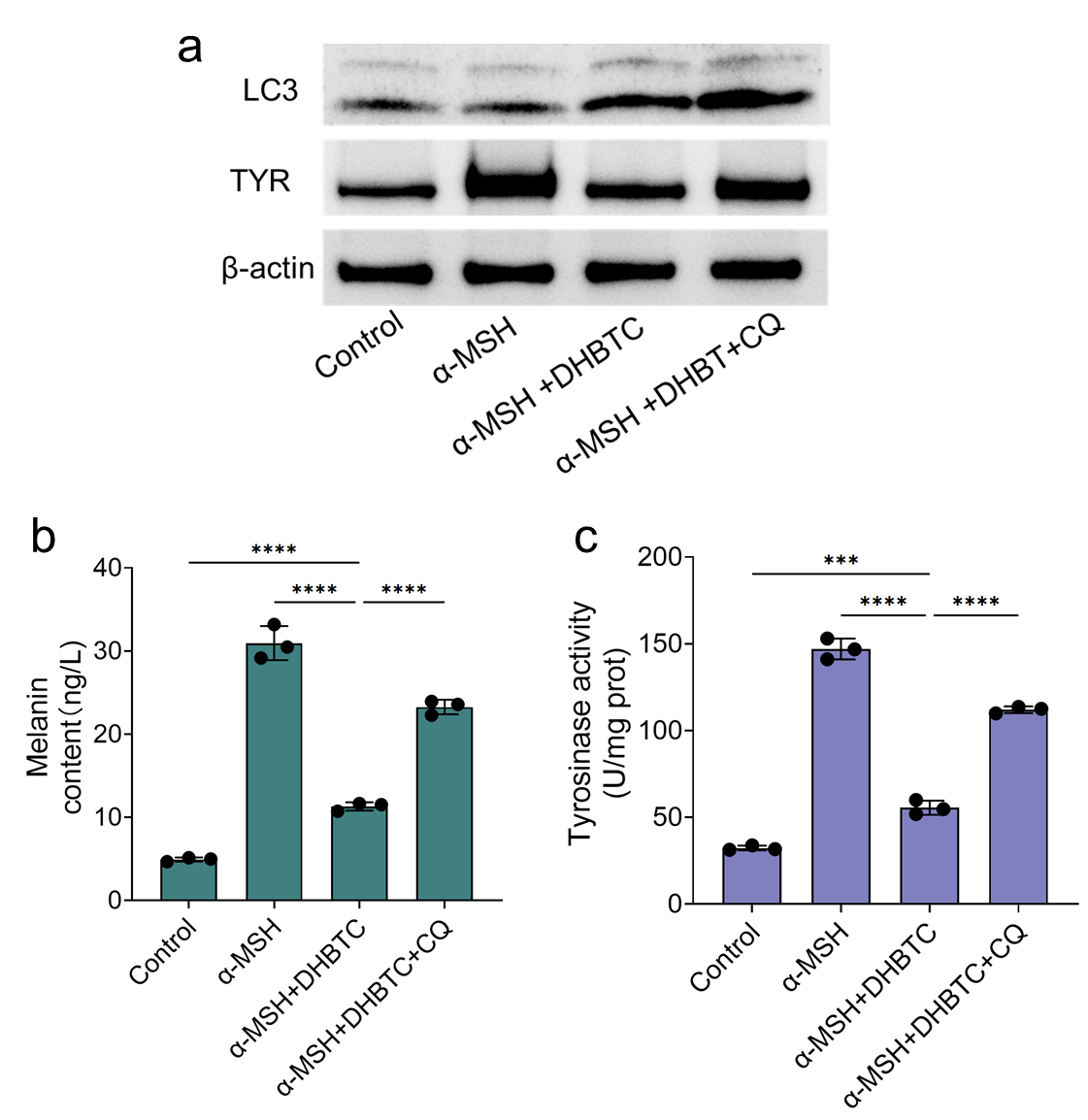


**Figure S8.** **Lysosomal inhibition rescues DHBTC-induced hypopigmentation. a,** Western blot analysis of LC3B and TYR protein expression in B16F10 cells treated with DHBTC (0.1 mg/mL) for 24 h in the presence or absence of the lysosomal inhibitor chloroquine (CQ, 20 μM). **b,** Quantitative analysis of intracellular tyrosinase activity in different treatment groups. **c,** Quantitative analysis of melanin content in different treatment groups. Results are shown as the mean ± SD for n = 3; ns: no statistical difference,*p <0.05, **p <0.01, ***p <0.001, ****p <0.0001.


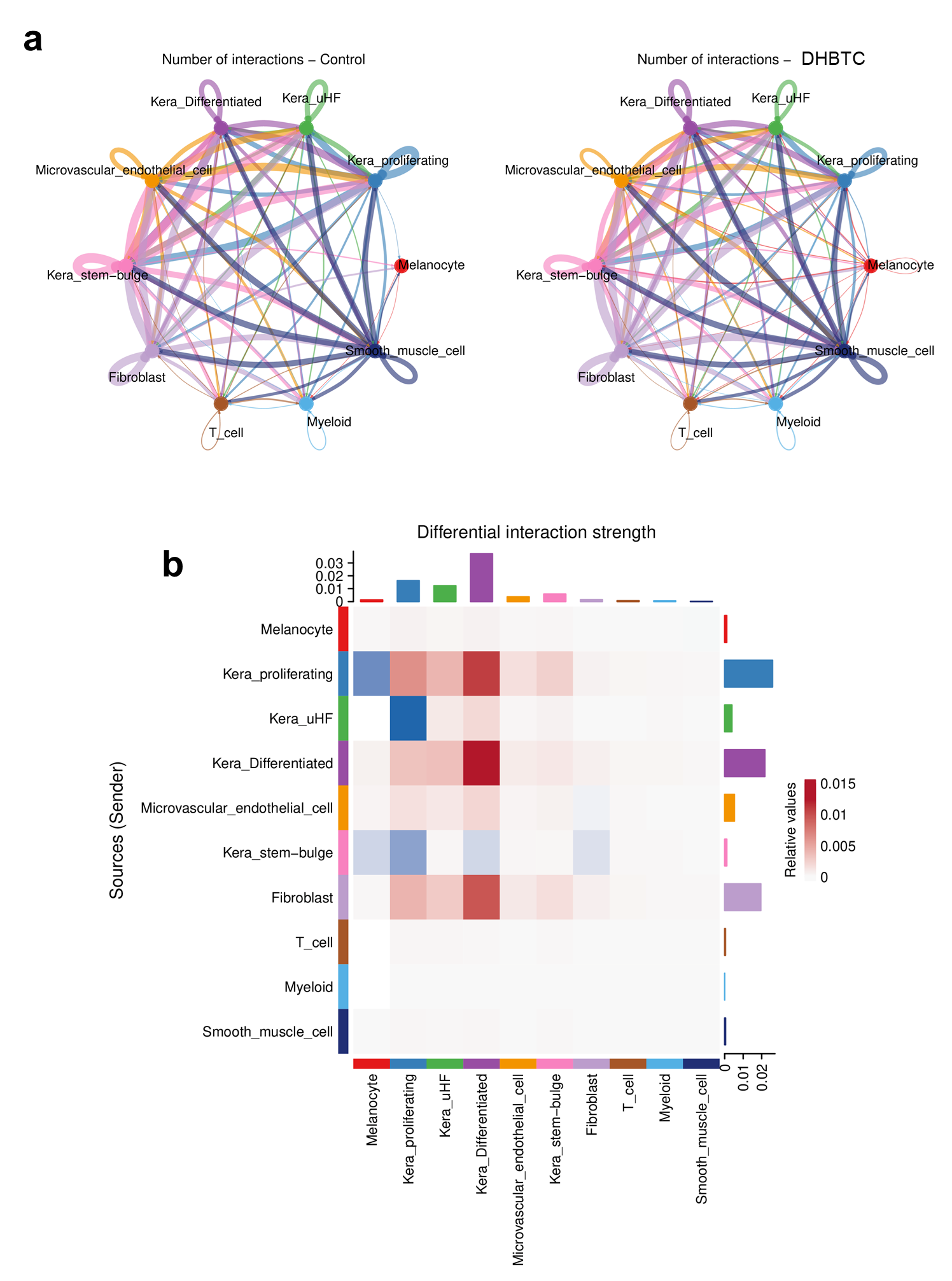


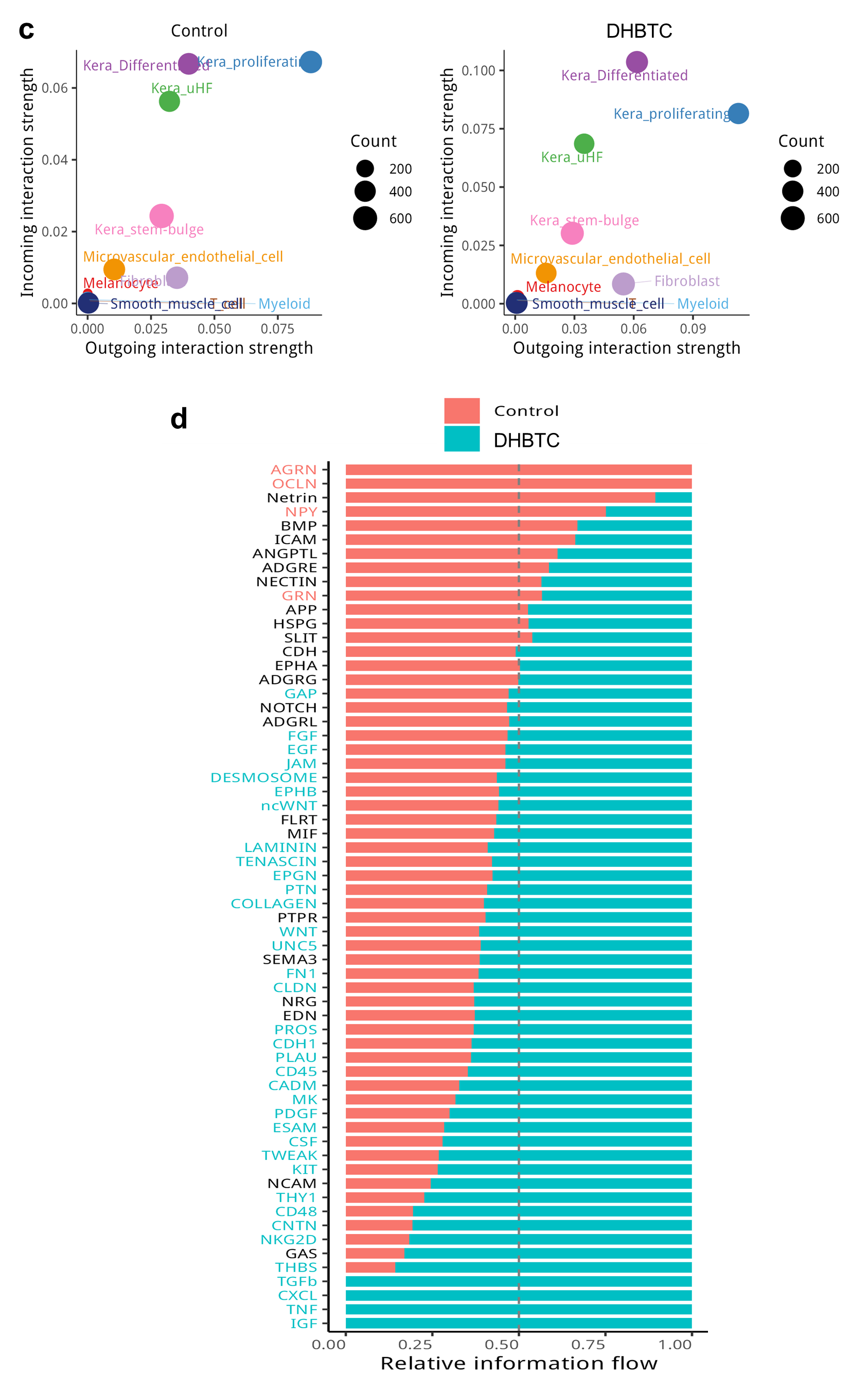


**
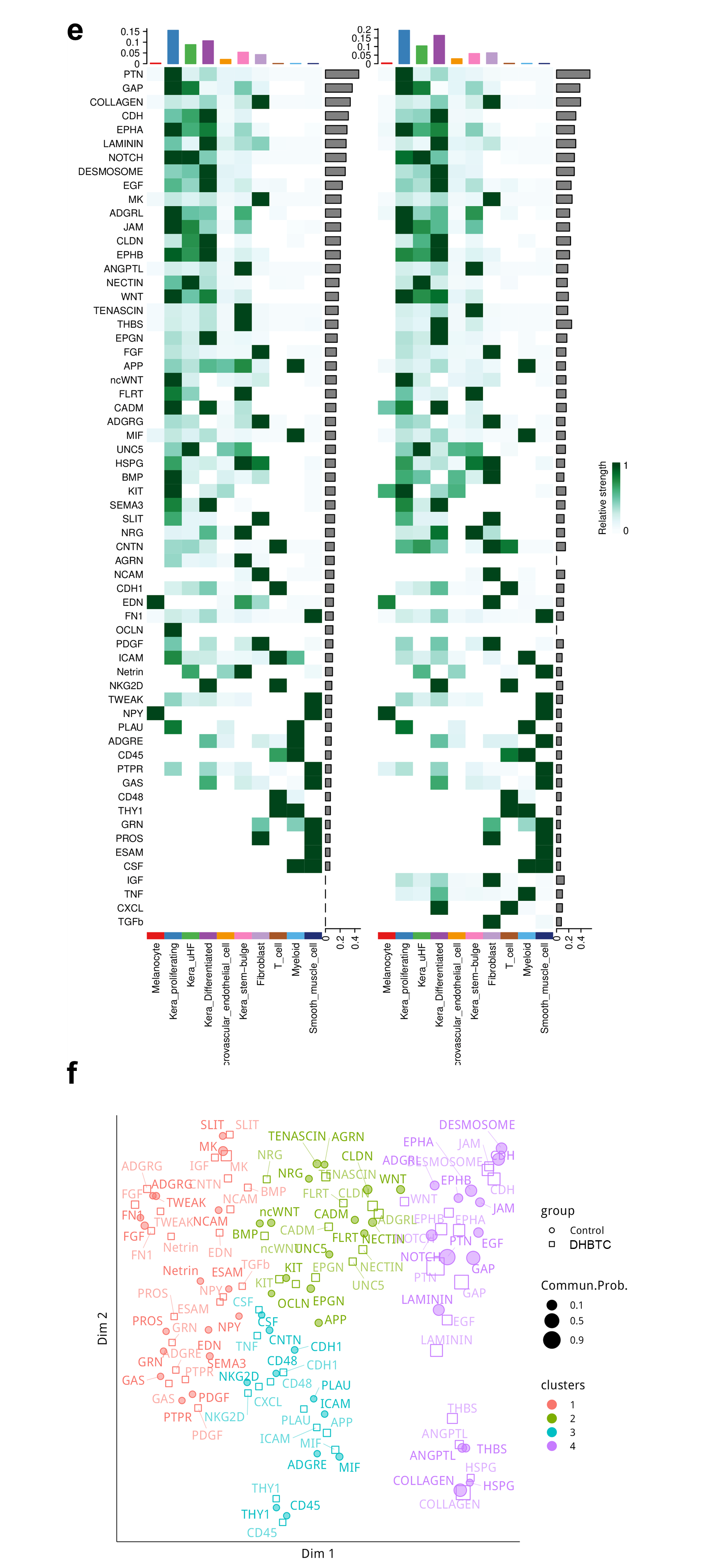
**

**Figure S9. a**. Chord plot of communication strength for different groups (left: cell population interactions for Control, right: cell population interactions for DHBTC). **b**. diffInteraction_weight_heatmap (Shows the difference in communication probability between ligand-receptor pairs across all cell subpopulations between the two groups. Blue lines indicate stronger communication for Control, red lines indicate stronger communication for DHBTC). **c**. signalingRole_scatter (Compares outgoing and incoming interaction strengths in two-dimensional space. Horizontal axis: outgoing interaction strength; vertical axis: incoming interaction strength; dots represent different cell populations). **d**. Information flow (Significant signaling pathways are ranked by information flow differences; each row represents a cell communication pathway. Significant signaling pathways are ranked based on the difference in overall information flow between Control and DHBTC within the inferred network). **e**. signalingRole_heatmap_all (Left: Heatmap of cell populations and signaling pathways corresponding to the Control group; Right: Heatmap of cell populations and signaling pathways corresponding to the DHBTC group). **f**. EmbeddingPairwise graph (the output of this result is calculated based on functional similarity. The colors of different points in the graph represent different groups, the different shapes of the points represent the signal pathways of different groups, and the size of the points is proportional to the communication strength).


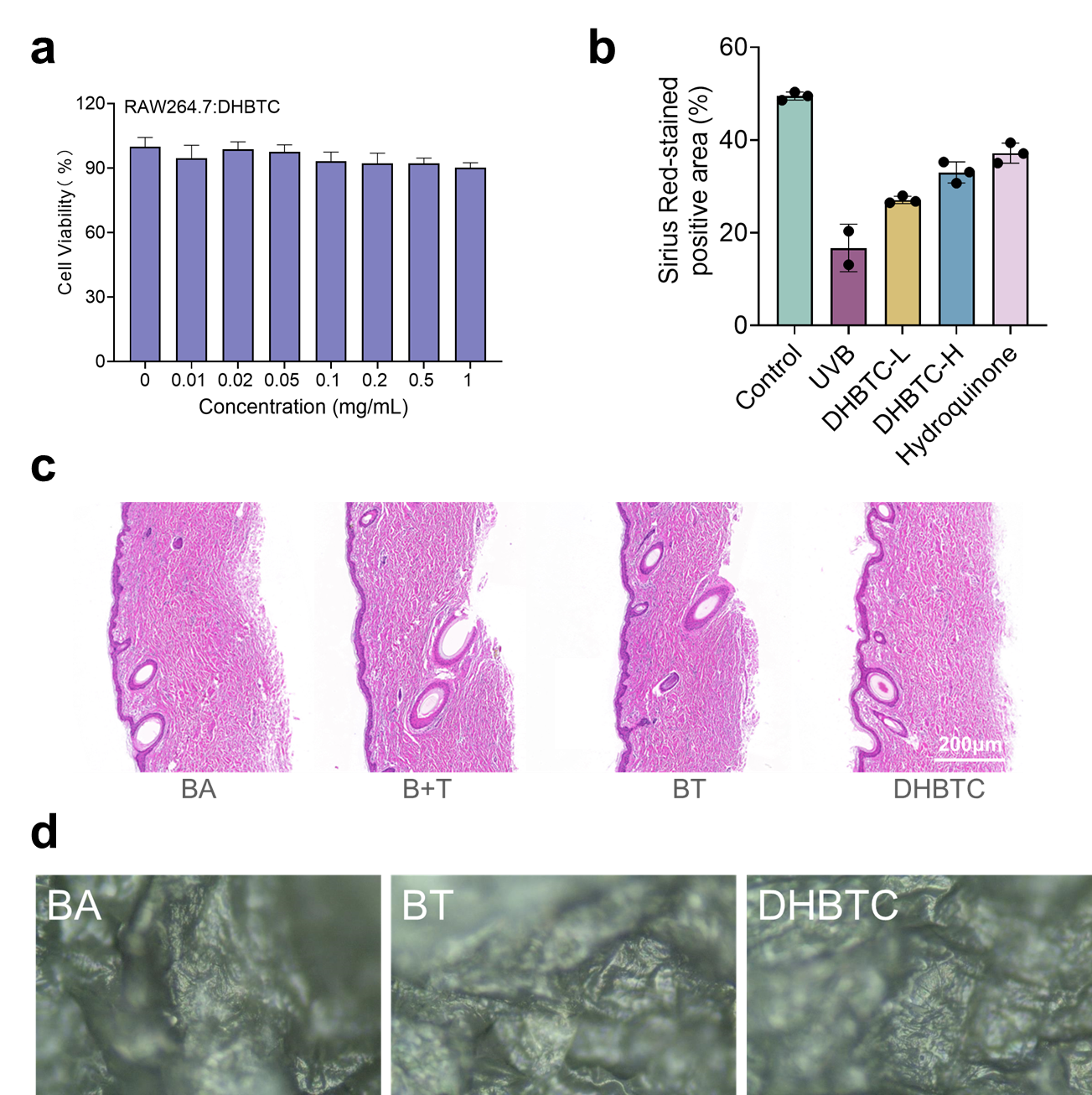


**Figure S10. Biosafety Assessment of the DHBTC System. a**, Cytotoxicity. **b,** Semi-quantitative results of Sirius Red staining. **c,** Representative images of hematoxylin and eosin (H&E)-stained porcine skin tissue from different treatment groups. Scale bar = 200 nm. **d,** In situ optical micrographs of the skin surface captured via the confocal Raman microscope system after treatment with BA, BT, and DHBTC.

**Table S1**. ^1^H NMR chemical shifts of BT and TA.

| Proton | δfree(ppm) | δionic salt(ppm) | ∆δ=δionic salt−δfree(ppm) |
| --- | --- | --- | --- |
| H-a | 2.73 | 2.81 | 0.08 |
| H-b | 1.99 | 2.12 | 0.13 |
| H-c | 1.81 | 1.89 | 0.08 |
| H-d | 1.53 | 1.60 | 0.07 |
| H-e | 1.27 | 1.34 | 0.07 |
| H-f | 0.95 | 1.01 | 0.06 |

**Table S2.** **Comparison of Physical and Chemical Properties of BT at Different Molar Ratios**

| **Group** | **Size** | **PDI** | **Zeta** | **Conductivity** |
| --- | --- | --- | --- | --- |
| BT=1:1 | 156.48 | 0.277 | -3.0 | 0.204 |
| BT=1:2 | 256.08 | 0.07 | -38.1 | 0.083 |
| BT=1:3 | 278.1 | 0.221 | -35.4 | 0.064 |
| BT=2:1 | 210.3 | 0.297 | -3.8 | 0.234 |
| BT=3:1 | 161.8 | 0.138 | -0.4 | 0.346 |

**Table S3. Comparison of Physical and Chemical Properties Between BT and DHBTC**

| **Group** | **Size** | **PDI** | **Zeta** | **Conductivity** |
| --- | --- | --- | --- | --- |
| BT | 156.48 | 0.277 | -3.0 | 0.204 |
| DHBTC | 196.04 | 0.265 | -6.1 | 0.177 |
